# Exploring the role of advanced oxidation processes in microplastics pollution research: A systematic literature review

**DOI:** 10.1007/s11356-026-38028-9

**Published:** 2026-07-13

**Authors:** Kaique Carvalho da Silva, Thiago Moreira de Rezende Araújo, Walter Ruggeri Waldman, Maria Cristina Canela

**Affiliations:** 1 Laboratório de Ciências Química, Universidade Estadual do Norte Fluminense Darcy Ribeiro, Av. Alberto Lamego, 2000, Campos Dos Goytacazes-RJ, 28013-602 CP Brasil; 2 Instituto Federal de Educação Ciência e Tecnologia Fluminense, Campus Campos-Centro, Dr. Siqueira, 273, Campos Dos Goytacazes-RJ, 28030-130 CP Brasil; 3 Centro de Ciências e Tecnologias para a Sustentabilidade, Universidade Federal de São Carlos, Rodovia João Leme Dos Santos, Km 110 - SP-264, CP, Sorocaba, 18052-780 SP Brasil

**Keywords:** Microplastics, Advanced oxidation processes, Aging, Degradation, Digestion, Adsorption, Desorption

## Abstract

Advanced oxidation processes (AOPs) are a promising tool for microplastics (MPs) pollution research, with applications in aging, degradation, digestion, adsorption, and desorption processes. Therefore, this review aimed to identify the main applications of AOPs and the findings from MPs studies. The systematic literature review was conducted using the Web of Science database, which retrieved 388 articles between 1990 and November 2nd, 2025. After screening and eligibility analysis, 84 articles were deemed eligible for inclusion in the final review. Fenton agents, H_2_O_2_, and their combinations were predominant in studies on MPs aging, degradation, and digestion, accounting for approximately 89.2% of the studies. Persulfate was the most effective oxidizing agent for aging, rapidly causing morphological and chemical modifications, including surface cracks averaging 206 nm. In contrast, degradation of highly resistant MPs requires more robust technologies to achieve 95.9% degradation in a hydrothermal process with lower energy consumption. In digestion protocols, NaClO achieved 88–92.1% organic matter removal while causing less damage to MPs. However, recovery rates revealed challenges with PVC and PET particles (24 – 93%), varying substantially with the type of matrix, polymer, and oxidant. The AOPs can also modify the surface of MPs, increasing the number of functional groups and altering their interactions with pollutants. Overall, this review demonstrates that although AOPs are promising strategies for the reviewed application areas, significant challenges remain regarding process optimization, energy efficiency, and large-scale environmental applications.

## Introduction

The global production of plastic has already reached 413,8 million tons annually by 2023, and the projections indicate an increase among 589 to 735,4 million tons in 2050 (Dokl et al. 2024; Thiumsak 2025; Statista 2025). The exuberant consumption of these products in contemporary society is a consequence of replacing traditional materials like glass and wood with more versatile, cheaper products and to satisfy market demands. This raises concerns among scientists, non-governmental organizations, and environmentally conscious citizens. Unfortunately, it’s expected that consumption of plastic products and, consequently, the generation of this type of waste will follow global population growth trends, reaching 1 billion tons of waste by 2060, with the environment as the final destination (OECD 2022; Olivatto et al. 2018).

The prolonged exposure of these materials to environmental degradation is characterized by photo-oxidative reactions that can alter the structures of polymer chains, thereby modifying the properties of plastics. This degradation process results in the formation of fragments with different sizes, including macro (> 1 cm), meso (1 cm – 5 mm), micro (5 mm – 1 nm), and nano (1 nm – 0.1 µm) particles (Olivatto et al. 2018). Microplastics (MPs) are emerging and persistent contaminants, defined by the National Oceanic and Atmospheric Administration (NOAA) as fragments, microbeads, plastic filaments, or any other plastic particles smaller than 5 mm (Arthur et al. 2009; Olivatto et al. 2018). The first documented reports of these contaminants date back to 1960 and 1972 (Campos da Rocha et al. 2021; Olivatto et al. 2018), when plastic particles were identified in the stomachs of seabirds (Bennett 1960) and in surface waters of the Sargasso Sea located in the North Atlantic (Carpenter and Smith 1972). Carpenter and Smith (1972) reported a concentration of 3,500 particles per square kilometer, most of which were white, pellet-shaped, with diameters ranging from 0.25 to 0.5 cm, and showed signs of degradation. As can be seen, it’s an old problem that has gained prominence in the last two and a half decades (Thompson et al. 2024). This growing attention is a result of the established term "microplastics" in the academic field in 2004 by researcher Richard Thompson, a professor of marine biology at the University of Plymouth, UK, when he quantified the abundance of MPs in beach and estuary sediments around Plymouth, UK (Thompson et al. 2004).

Classification of MPs by origin is another way to determine the source of contamination. Primary MPs are manufactured with predefined shapes and sizes (< 5 mm) to primarily meet the demands of the cosmetics industry, thereby contributing significantly to micro- and nanoplastic (NPs) contamination. Secondary MPs are particles resulting from the degradation process (whether discarded waste or primary materials like synthetic clothing and materials still in use) that accumulate in the environment and undergo mechanical and UV light radiation, breaking down into smaller particles (Montagner et al. 2021; Osman et al. 2023; Sodré et al. 2023; Vargas et al. 2022). The MPs are ubiquitous pollutants that accumulate in the environment in different matrices such as surface and deep waters, sediments, soils, air, and living organisms. This raises considerable ecological concern, since microplastics not only bioaccumulate but also act as vectors for other pollutants such as pesticides, toxic metals, persistent chlorinated and aromatic compounds, amplifying risks to ecosystems and human health (Barceló et al. 2023; Olivatto et al. 2018; Westphalen and Abdelrasoul 2018). Evidence of these particles already exists in both aquatic ecosystems near large urban centers and in uninhabitable regions of the planet, underscoring the importance of actions to curb their global distribution (Wright and Kelly 2017; Campos da Rocha et al. 2021).

## Photodegradation mechanisms of microplastics in the environment

The exposure of plastic waste in the environment for long periods is marked by photo-oxidative reactions (Olivatto et al. 2018). The main results regarding the characteristics of the materials are described in the literature as cracking, erosion, discoloration, phase separation, molar mass loss, increased crystallization, oxidation, and changes in polymer wettability. This set of effects can pose numerous complex challenges for ecosystems and organisms, since, depending on the species of radicals and the type of polymers, various routes can be inferred to understand the phenomena (Montagner et al. 2021; Singh and Sharma 2008).

**Fig. 1:**
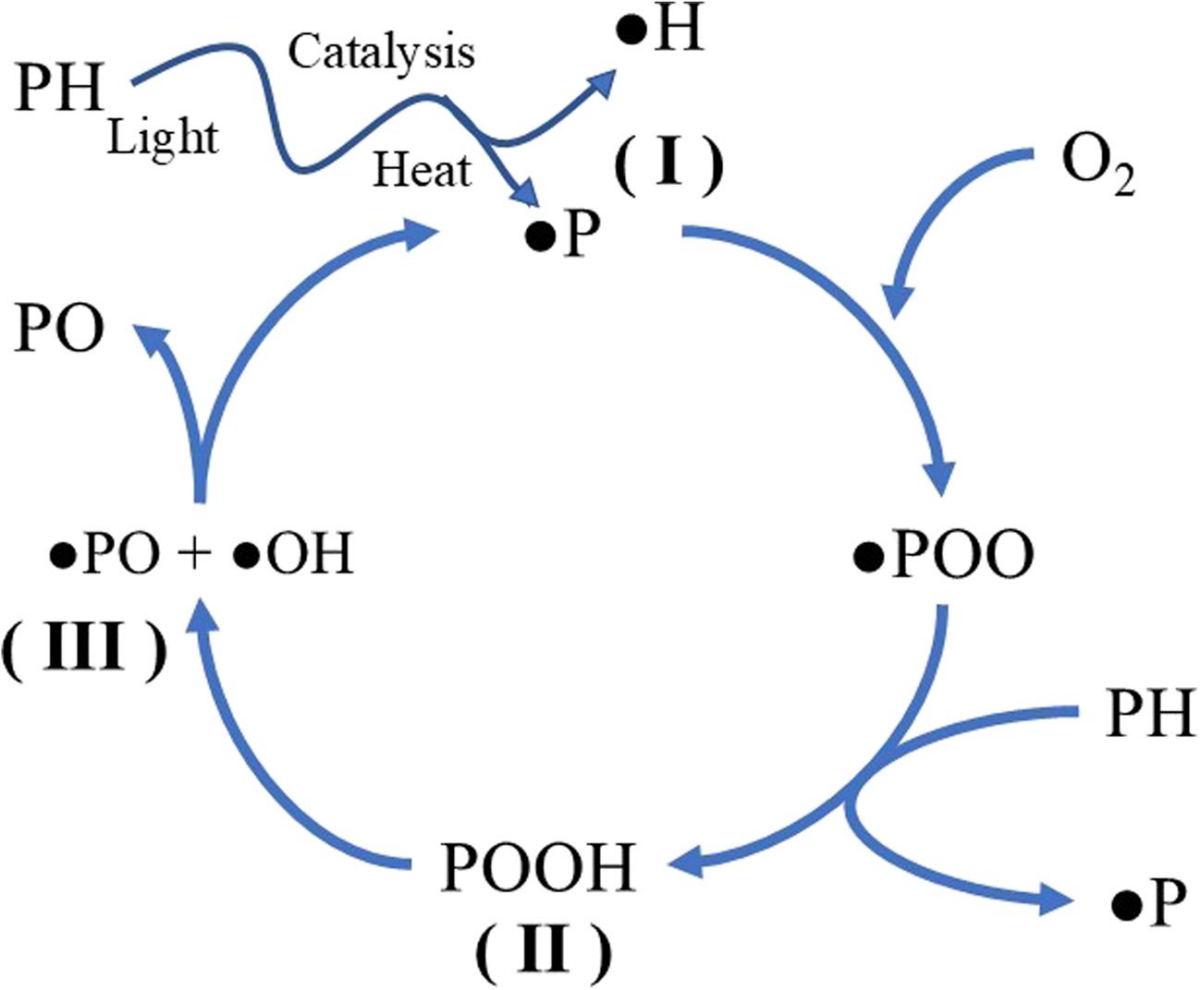
High catalytic cycle of alkoxy radical (•PO) formation (adapted from (Cacuro et al. 2018)

Photo-oxidative reactions are processes responsible for the degradation of plastic waste by light, a primary source of damage to polymeric materials and a direct driver of the production of secondary microplastics (Singh and Sharma 2008). Oxygen is an element present in the environment that plays an important role in oxidation reactions. This is particularly evident in polymers because this element participates in the catalytic reaction involves forming and breaking down hydroperoxides, the most significant compounds in the process of the catalytic cycle of alkoxy radical (•PO) (P = polymer) formation (Fig. 1). The radical •PO is responsible for breaking the polymer chain after plastics are exposed to temperature and UVA radiation in the presence of oxygen. This is one of the main mechanisms that modify their properties, making them more brittle and prone to forming MPs (Padron 1989). These radicals play a pivotal role in polymer degradation. (I) Upon exposure to UV radiation and thermal energy, the free radical (•P) is generated by homolytic cleavage of C–C and C-H bonds, redistributing the bonding electrons between the two resultant species, which then serve as initiation radicals. (II) In the presence of molecular oxygen (O₂), the radical (•P) facilitates the formation of hydroperoxide (POOH), one of the most important initiators of thermal and photochemical oxidation. (III) Subsequently decomposes to yield alkoxy (•PO) and hydroxyl (•OH) radicals. The alkoxy radical (•PO) undergoes further absorption of UV radiation, transforming into a carbonyl group and initiating the cleavage of the polymer chain (Cacuro et al. 2018; Singh and Sharma 2008).

Polymers are composed of amorphous domains, which are predominant in pristine materials, and crystalline domains, which are less abundant. The degradation process increases the breaking of polymer chains, resulting in greater chain mobility that can contribute to crystalline domain formation and the generation of polar groups such as hydroxyl (O–H) and carbonyl (C = O). Because of these modifications, the physical characteristics of plastics change, transforming materials that were once flexible, hydrophobic, and transparent into brittle, hydrophilic, and opaque ones (Cacuro et al. 2018; Waldman and Rillig 2020). To understand these characteristics and the behavior of pristine and degraded microplastics, and the degradation processes (Waldman and Rillig 2020), other advanced oxidizing agents have been employed further to investigate these mechanisms and their environmental behavior and fate.

## Advanced oxidation processes: fundamentals and oxidants

Advanced Oxidation Processes (AOPs) are based on the generation of strong and efficient oxidants to remove environmental pollutants, and their application for the disinfection and decomposition of water contaminants dates back many years. Production of reactive oxidizing species, such as hydroxyl radicals (•OH) and sulfate radicals (•SO_4_), which are potent oxidants capable of interacting with a wide range of organic compounds (Andreozzi et al. 1999; Vagi and Petsas 2020). In the context of plastic degradation, these radicals initiate structural modifications in polymer chains, leading to the formation of hydroxyl and carbonyl groups and altered C-H bonds (Rizwan and Bilal 2022).

Radical production can occur via various routes, using a wide range of substances as oxidizing agents and/or catalysts. AOPs are most classified into homogeneous and heterogeneous processes. Homogeneous AOPs use catalysts in gases or the liquid phase, while heterogeneous AOPs employ solid-phase catalysts, such as titanium dioxide (TiO₂) or other solid catalysts (Vagi and Petsas 2020). However, according to MPs research, the homogeneous group is the most common in the field. And some instances of homogeneous processes involve are: O_3_-based methodologies, which comprise O_3_, O_3_ + UV, O_3_ + H_2_O_2_, and O_3_ + UV + H_2_O_2_; wet peroxide oxidation, which utilizes H_2_O_2_ as the oxidizing agent and typically functions at temperatures below 373 K; persulfate oxidation with UV and heat activation (${S}_{2}{{O}_{8}}^{2-}$); Fenton-based methodologies, including the traditional Fenton process (H_2_O_2_ + Fe^2+^) (Ribeiro et al. 2015; Rizwan and Bilal 2022).

The ozone process is based on the behaviour of O_3_ in aqueous Alkaline solution. The short-lived nature of O_3_ is explained by its decomposition in aqueous solution, which proceeds via the formation of •OH. In ozonation, pollutants can be degraded by direct reactions with O_3_ and by indirect reactions with hydroxyl radicals. The reactive species are produced through photolysis (Eq. (1)), which initiates the dissociation of ozone and water, leading to their formation via subsequent chain reactions. The addition of H_2_O_2_ during ozonation is also a common practice, and increasing the pH of the solution is a possible strategy to improve the oxidizing power of the system (Eq. 4) (Andreozzi et al. 1999; Ribeiro et al. 2015). In addition to being an oxidant, H_2_O_2_ decomposes upon irradiation, forming oxidizing radicals. The homolytic cleavage of H_2_O_2_ in this process involves the breaking of the O–O bond, responsible for the formation of two •OH (Eq. 5) (Andreozzi et al. 1999).

1 $${O}_{3}+hv \to 2 \cdot OH$$

2 $$O_3+H_2O_2\rightarrow\bullet\;\,\,OH+2O_2+\bullet\;OH_2$$

3 $$H_2O_2+hv\rightarrow2\bullet OH$$

The combination of H_2_O_2_ with catalyst species, identified by Fenton in the previous century, is a classic reactive system that continues to attract substantial research focused on its usefulness in methodologies for degrading a wide variety of compounds. (Andreozzi et al. 1999). The use of hydrogen peroxide (H_2_O_2_) and divalent iron catalyst species (Fe^2+^) proceeds at room temperature and pressure, under acidic pH values of 2.5–3.0, and requires no special equipment or energy to activate H_2_O_2_. The specific oxidation treatment proceeds by the decomposition of H_2_O_2_ molecules into •OH, as shown in Eq. (6) (Vagi and Petsas 2020).

4 $${Fe}^{2+}+{H}_{2}{O}_{2}\to {OH}^{-}+\cdot OH$$

The persulfate anion has been newly used as a powerful oxidizing agent, showing effective results in treating water and soil contaminated with organic pollutants. With high solubility, stability, and low cost, it makes it a valuable source of oxidizing radicals. Unlike typical AOPs, where •OH is the main oxidant, activated persulfate also forms sulfate radicals (•SO₄⁻), which have a higher oxidation potential. Persulfate activation, essential for effective pollutant degradation, can be achieved via physical, chemical, or metal-catalyzed methods, with homogeneous catalysis with temperature and UV radiation (Eq. (7)) being the most commonly applied (Zhou et al. 2018).

5 $${S}_{2}{{O}_{8}}^{2-}+\frac{Heat}{hv}\to \cdot {{SO}_{4}}^{-} +\cdot OH$$

Despite the substantial increase in microplastic research in recent years, significant uncertainties remain regarding sample preparation protocols and the effectiveness of current removal strategies. In this context, AOPs have emerged as a promising tool for developing MP studies, supporting application such as aging (Lang et al. 2020; Liu et al. 2019; Ouyang et al. 2022; Wang et al. 2020), degradation (Easton et al. 2023; Wang et al. 2023b; Zhang 2020), digestion sample (Duan et al. 2020; Ge et al. 2024; Tan et al. 2022) and adsorption (Ye et al. 2020; Zhang et al. 2023a). These processes help eliminate organic and biological matter in complex matrices, reducing analytical bias and improving the performance of characterization techniques such as microscopy, µ-FTIR-ATR, and µ-Raman (Fernandes et al. 2022; Soursou et al. 2023). Moreover, the use of AOPs for MPs degradation has become increasingly relevant, as conventional water and wastewater treatment systems exhibit limited efficiency in removing these contaminants (Rizwan and Bilal 2022). Thus, the objective of this literature review is to examine the main applications of AOPs and to analyze the findings from studies on aging, degradation, digestion, adsorption, and desorption of MPs. Verify how often the application of pristine and degraded MPs, and the chemical and physical characterization methods were used in the AOPs applications.

## Literature review

A systematic bibliographic search was conducted on the Web of Science platform. In order to obtain a comprehensive range of relevant publications during the identification phase, keywords and topics were strategically interconnected using the Boolean operators "AND," "OR," and "NOT" within sophisticated search mechanisms. The keywords and topics employed in the search of titles and abstracts were:

- (Title) microplastic* AND;
- (Title) “Fenton OR persulfate OR AOP OR oxidation OR digestion OR H2O2 OR enzym* OR acid OR basic OR alkaline OR KOH OR NaOH”;
- (Topic) AND “validation OR study OR stability OR degrad* OR pristine OR virgin.”
- The topics and categories excluded from the search were:
- (Web of Science Categories) NOT Biotechnology & Applied Microbiology NOT Neurosciences;
- (Topic) NOT microbial OR biodegradation.

The search covered different matrices, including ultrapure water, surface water, sludge, fish, and simulated samples containing organic matter (humic acid). The investigation also encompassed a range of research objectives, including adsorption/desorption, aging, and degradation. Some actions were necessary to emphasize bibliographic research and identify possible applications of AOPs in studies related to MPs. Keywords were selected to retrieve the maximum number of relevant publications. Biodegradation and microbial degradation topics were excluded because these processes are not points of interest for the research. The eliminated topics produced outcomes that significantly diverged from the intended focus of the review; terms such as microplastic, plastic, digestion, acid, and enzymes are used without the context of digestion processes with AOPs in polymers. The fields of Biotechnology and applied microbiology use the term "Plasticity" to refer to the inherent capacity of organisms or biological systems to adapt and modify in response to varying conditions or environmental stimuli, often leading to alterations in structure, function, or behavior. In Neuroscience, however, the term denotes the capability of the nervous system, particularly that of the brain, to adapt and reorganize in response to alterations in experience, environmental factors, and learning processes. In Polymer Science, this terminology pertains to the deformation and molding characteristics of polymeric materials, which are associated with their capacity to be permanently reshaped, elongated, or altered under the influence of forces, thermal conditions, or chemical transformations in their structure, a focal point of this investigation. This exemplifies a term that may complicate the identification of pertinent scholarly works and elucidates the necessity of excluding such categories from the search tool.

The bibliographic search yielded 388 records from 1990 to November 2, 2025 (date of the search). The records were exported to the Rayyan web app for data management and evaluation. To include articles in a systematic search, specific criteria were established:

- Using AOPs, especially to degrade, age, extract microplastics, or digest organic matter contained on their surface, as well as evaluating changes in microplastics caused by using AOPs with the support of spectroscopic and microscopic techniques.

The exclusion criterion was:

- Use of acidic, alkaline, and enzymatic digestion methods.

**Fig. 2:**
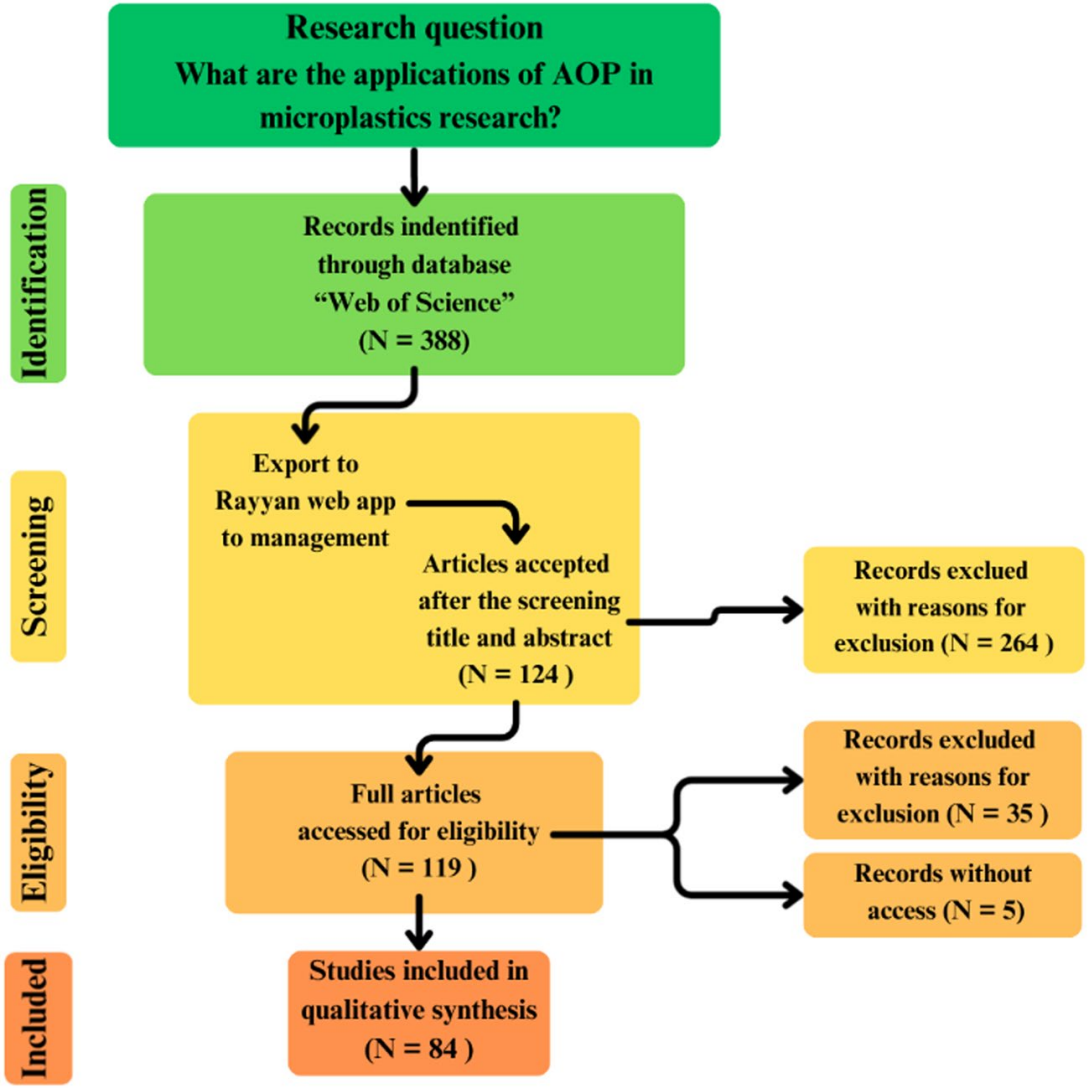
Flow diagram of each stage of the literature search

Title and abstract screening were conducted in the Rayyan web app, and the initial articles met the established inclusion and exclusion criteria, allowing them to proceed to the following eligibility stage. Unfortunately, 264 articles did not meet the criteria and were excluded. During the eligibility stage, 119 articles were accessed and evaluated, however, five were inaccessible. After careful consideration, 84 articles were included for study, and 35 were excluded based on predefined criteria. Figure 2 presents a flowchart outlining each stage of the bibliographic search along with the corresponding results.

## Results and discussion

Following a depth analysis of the 84 articles (100%) included in the review, several general topics emerged regarding the application of AOPs in research focused on MPs. The methodologies involving UV radiation, Fenton's reagent, persulfate, Hydrogen Peroxide (H_2_O_2_), and various H_2_O_2_ derivatives are the most predominant strategies in studies of MPs. UV radiation and persulfate are common oxidizing agents to induce the aging of MPs. These oxidative methods can simulate the natural degradation of polymers under controlled conditions, enabling investigations into the degradative behavior of various polymers in the environment and their interactions with organic matter and persistent contaminants (Liu et al 2019). The Fenton and H_2_O_2_ methods are commonly employed in investigations focused on detecting and quantifying MPs in surface waters, sediments, and biota (Soursou et al. 2023), with the application of each oxidizing agent directly linked to the matrix being investigated. H_2_O_2_, recognized as a mild oxidant and widely employed for the degradation of tissues in biota samples (Fernandes et al. 2022), has demonstrated better performance for this type of organic matter than others oxidants. NOAA recommends implementing Fenton oxidation for surface water and sediment samples. This approach aims to oxidize the organic matter present, thereby enhancing the chemical identification of plastic particles (Arthur et al. 2009; Campos da Rocha et al. 2021).

**Fig. 3:**
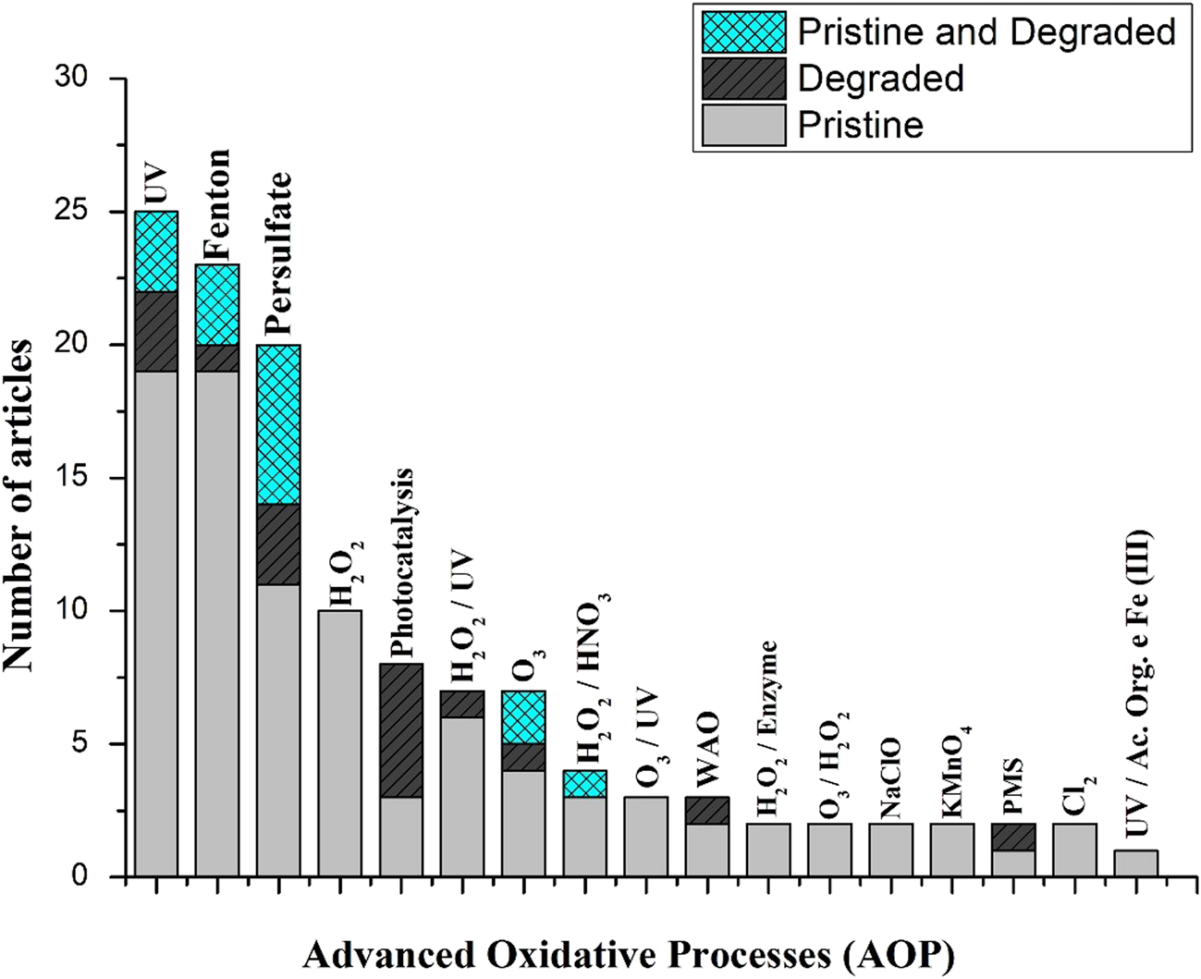
Number of articles for the different sets of AOPs used and the level of degradation of the microplastics

These oxidative methods have been frequently applied in microplastic studies for different purposes and matrices. In many cases, they are used to compare the best results and assess the impact of these processes on the physical and chemical characteristics of the polymers. Among the studies included in the literature search, it was notable that pristine microplastics were the most used, and only 19 articles (22.62%) used microplastics that were degraded or both degraded and pristine. Another relevant observation is that half of these studies with degraded particles were only published in the last 2 years. The predominant use of pristine microplastics in most studies underscores the need for further research on degraded microplastics, which exhibit different characteristics from pristine ones (Waldman and Rillig 2020). Figure 3 illustrates the number of articles and the microplastic degradation level used in each AOP method.

**Fig. 4:**
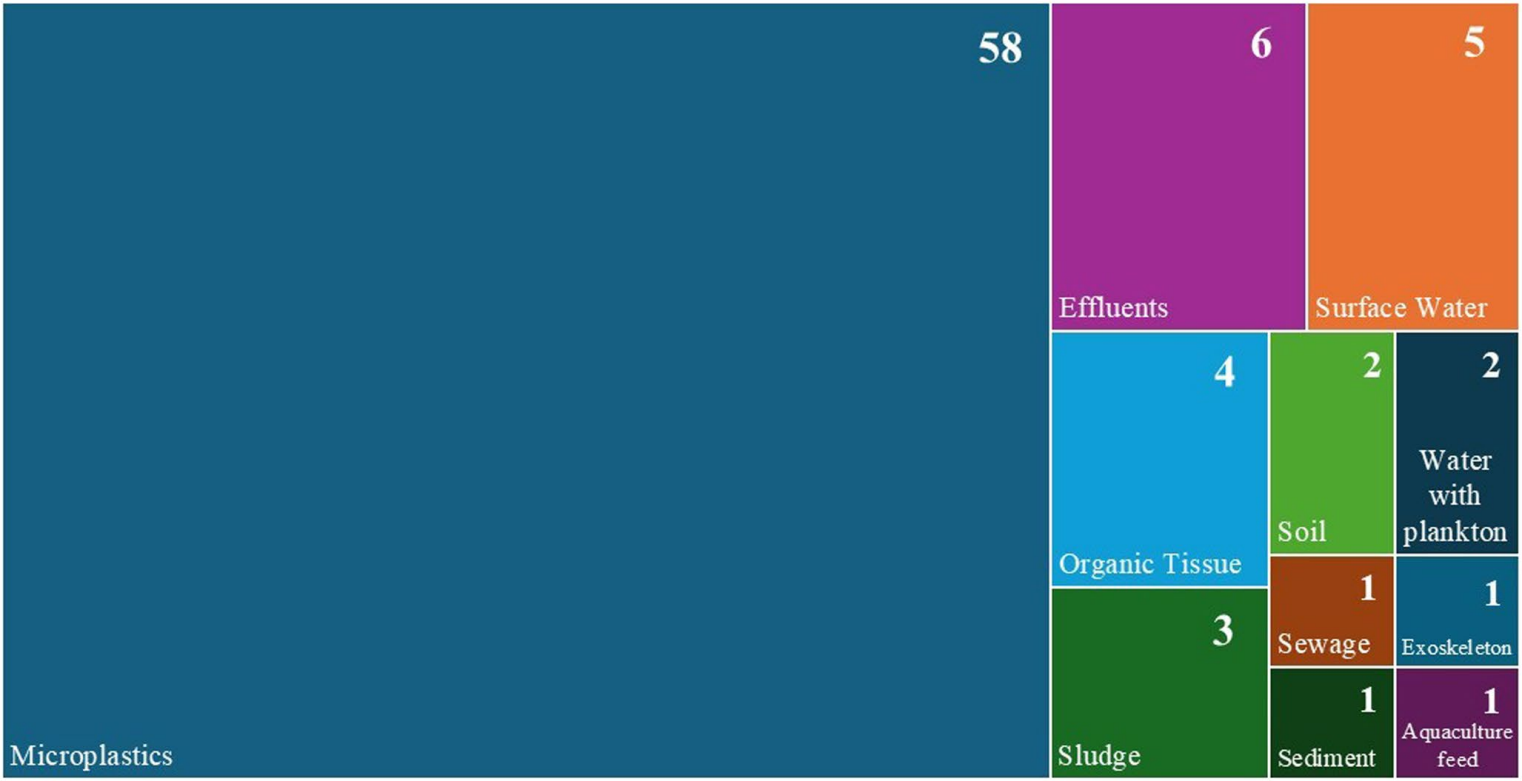
Distribution of articles as a function of the studied matrices

The primary application matrix of AOPs in our reviewed literature is microplastics, as shown in Fig. 4. The evaluation of structural changes in these plastic particles was reported in 58 (69.0% of the total) of the analyzed studies. Aging processes are the most common application of AOPs (41.6% of the total). Ultraviolet (UV) radiation is frequently used to simulate the effects of solar radiation on MPs and evaluate their aging behavior. However, persulfate and ozone also emerge as significant alternatives, being applied in 12 (14.3% of the aging articles) of the articles to investigate the aging of MPs from everyday materials. These oxidizing agents offer the advantage of faster reaction rates than UV processes, thereby accelerating this type of study. The oxidative generation of powerful radicals and the high water solubility are desirable characteristics for the development of research on MPs. These processes are responsible for the formation of microcracks in polymers, altering their physical and chemical properties. Consequently, properties such as absorption, colloidal stability, color, and fluidity of these materials expand our understanding of the environmental effects and risks associated with MPs in ecosystems (Lang et al. 2020).

The remaining combined matrices accounted for 26 articles (31,0% of the total) and were characterized by high organic matter content and distinct characteristics. Wastewater and surface water are the main matrices studied, with organic matter digestion and recovery protocols being the main applications (21.4% of the total). The removal of organic matter is an essential step in any methodology aimed at assessing microplastic contamination in the environment, as its presence interferes with the chemical identification of MPs. The extraction of these particles is also a crucial challenge because, depending on the type of PM, they tend to become trapped in organic matter and float, leading to underestimated results (Savino et al. 2022).

The considerable number of publications over the past ten years has raised questions among researchers about analytical procedures for microplastic analysis in environmental matrices, prompting calls to standardize approaches (Thompson et al. 2024). Studies on the efficiency of AOPs, the impacts on microplastics during the organic matter digestion step, and how these factors affect results have become more common. Soon, this may become a requirement for publications. Studies on the recovery of microplastics represent nine articles and cover samples from effluents, drinking water, soil, soft tissue, sludge, exoskeletons, and aquaculture feed. Furthermore, the growing diversity of research underscores the importance of interdisciplinary collaboration among scientists to address the complexities of microplastic pollution. The development of standardized methodologies across matrices ensures comparability across studies and improves understanding of the consequences for biodiversity and human health.

**Fig. 5:**
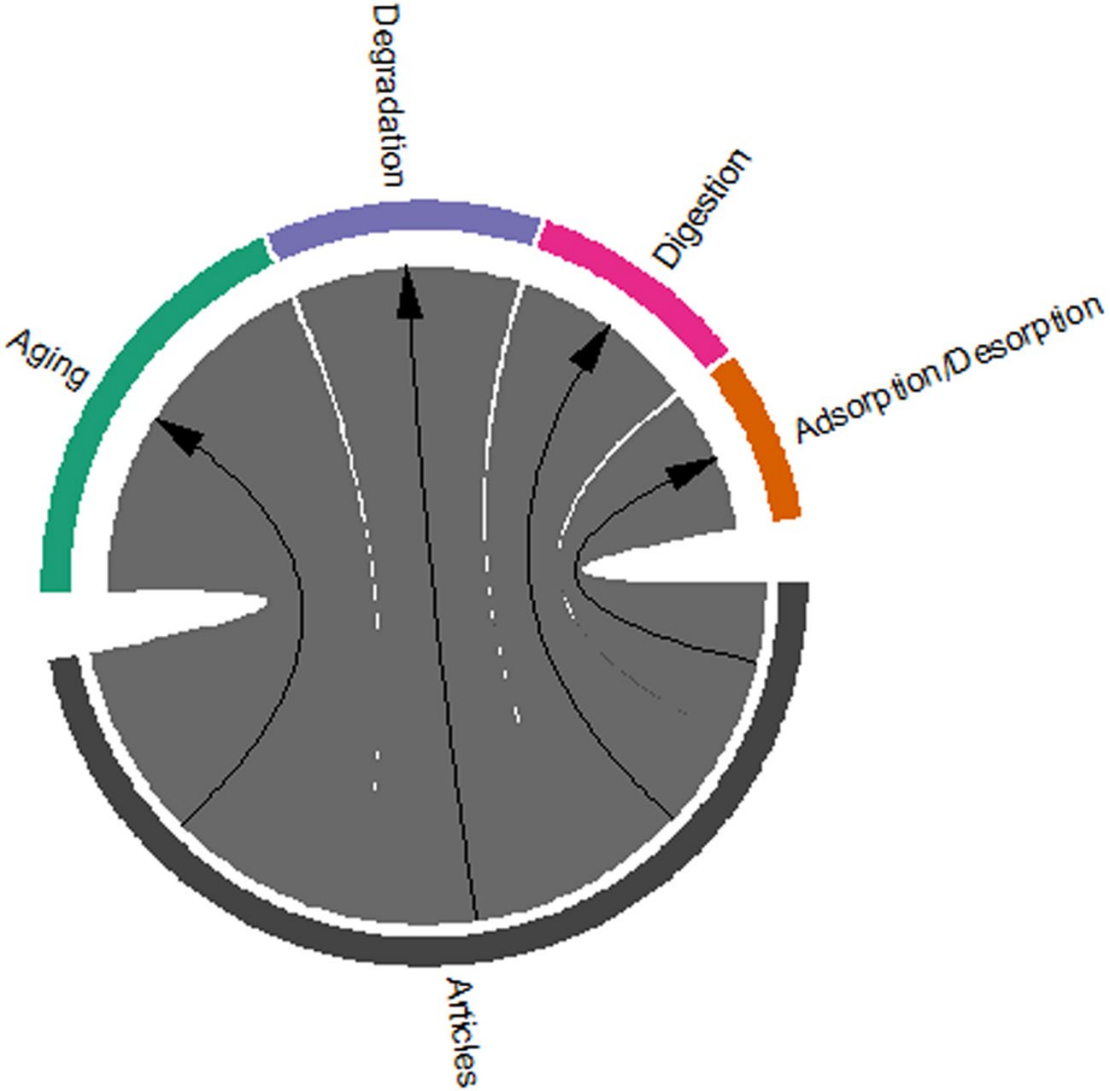
Graph of AOPs applications found in selected studies

In this context, aging (41.6%), degradation (26.2%), and digestion (21.4%) have emerged as the predominant areas of investigation, accounting for approximately 89% of the studies analyzed, as shown in Fig. 5. The degradation processes of MPs aim to eliminate plastic particles by evaluating mass reduction after AOPs application. Wastewater treatment plants (WWTPs) are a significant source of microplastic contamination, especially when their effluent is discharged into water bodies. Conventional treatment methods are largely ineffective in achieving complete mineralization of these particles. Although 75–99% of MPs are retained during coagulation and subsequently accumulate in sludge, smaller particles can readily pass through physical treatment stages (Wang et al. 2023a). Despite this high retention rate, the sheer volume of treated wastewater results in a substantial release of MPs into the environment (Belé et al. 2021). Traditional oxidative processes, such as Fenton reactions, typically yield low mass loss (Ortiz et al. 2022; Zhou et al. 2022). However, hydrothermal processes combined with Fenton demonstrate higher efficiency, with mass loss and complete mineralization (Wang et al. 2023a; Wang et al. 2023b). This approach appears to be a promising pathway to new studies and, consequently, to reducing the release of particulate matter into the environment.

The processes of desorption and adsorption of compounds on the surface of MPs represent the least-studied category, accounting for only 16.6% of the analyzed publications. Plasticizers, stabilizers, flame retardants, and toxic metals are among the additives incorporated into plastic and are an integral part of their functional characteristics (Westphalen and Abdelrasoul 2018). The desorption of these compounds following MPs degradation is an important focus of investigation for elucidating this mechanism and its associated toxicity to ecological systems. In addition, persistent contamination, such as pharmaceuticals (Zhang et al. 2023a) and pesticides (Belé et al. 2021), can be adsorbed onto the degraded surfaces of MPs, thereby promoting their bioaccumulation and biomagnification and exacerbating the presence of these deleterious substances (Ye et al. 2020). Understanding the complex interaction between MPs and contaminants is essential for evaluating their impact on environmental health and for developing regulatory measures to address this problem. Ultimately, new studies examine not only the chemical properties of microplastics but also their physical traits and how these factors affect the movement and fate of various contaminants across different ecosystems (Menéndez-Pedriza and Jaumot 2020).

**Fig. 6:**
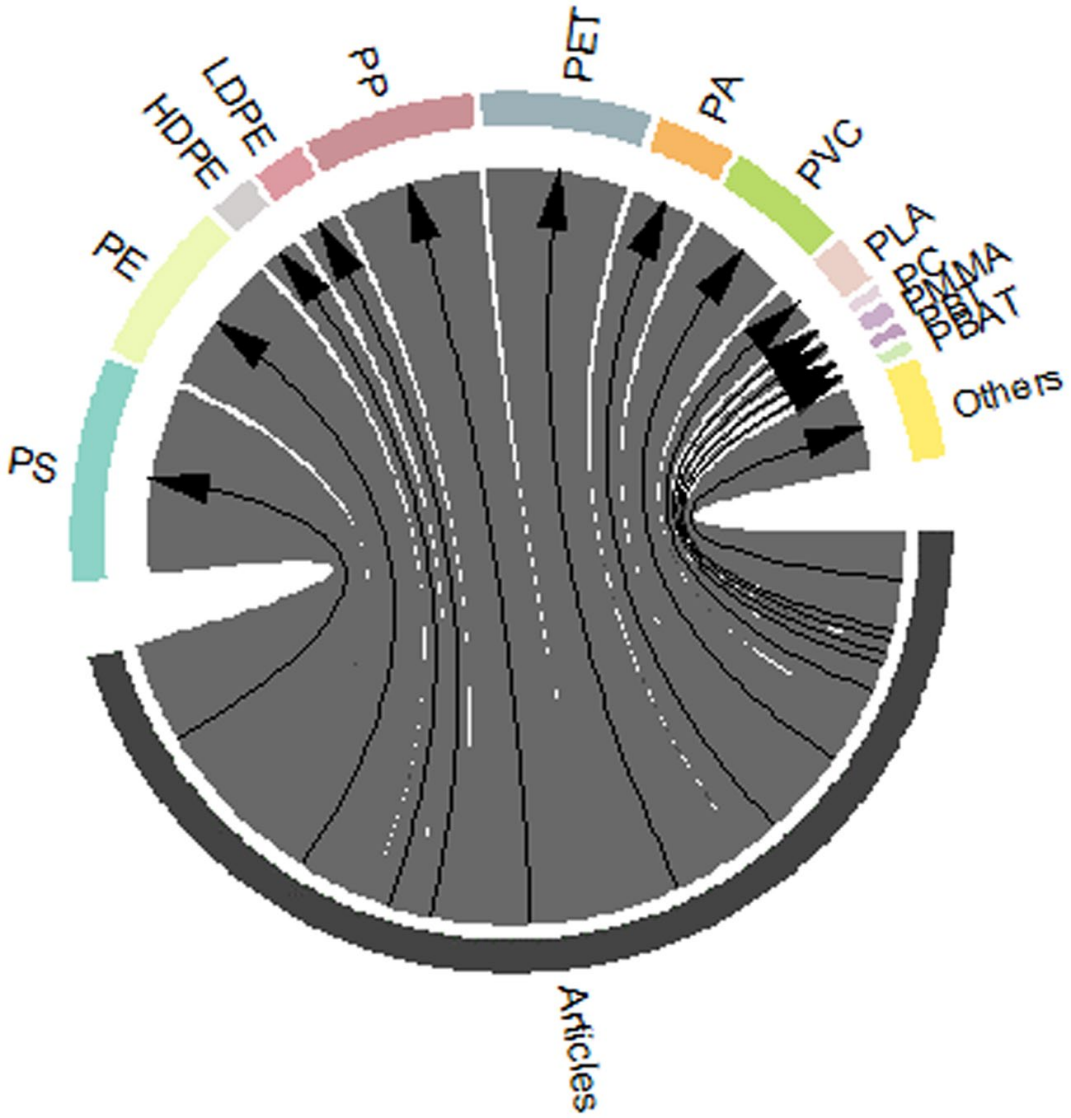
Graph of polymer types found in selected studies. **PVC**, polyvinyl chloride; **PA**, polyamide; **PET**, polyethylene terephthalate; **PP**, polypropylene; **LDPE**, low-density polyethylene; **HDPE**, high-density polyethylene; **PE**, polyethylene; **PS**, polystyrene; **PLA**, polylactic acid; **PC**, polycarbonate; **PMMA**, poly (methyl methacrylate); **PBT**, polybutylene terephthalate; **PBAT**, poly(butylene adipate-co-terephthalate)

Polypropylene (PP), polyethylene (PE), and polyvinyl chloride (PVC) are thermoplastic resins most predominantly used in the construction, food, and automotive sectors, including the manufacture of auto parts. Consequently, they contribute significantly to global waste production (Geyer et al. 2017; Montagner et al. 2021; Olivatto et al. 2018). The reviewed studies predominantly employed polystyrene (PS), polypropylene (PP), polyethylene (PE), and Polyethylene Terephthalate (PET) as representative MPs for the evaluation of AOP applications. Addressing the complexities associated with these materials requires a comprehensive understanding of their ecological interactions, effective eradication methods, and practical monitoring approaches, which may significantly help alleviate the repercussions of MPs mitigation. However, biodegradable polymers are understudied, with polylactic acid (PLA) being prominent among the selected studies. They are marketed as ecological solutions to mitigate environmental impact, but the PM particles from these materials continue to degrade in environmental conditions (Ge et al. 2025). Therefore, understanding the degradation mechanism of this type of polymer compared to non-degradable ones would be interesting and crucial for assessing their environmental fate and risks. Figure 6 provides a graphical overview of the kind of microplastic materials employed.

Spectroscopic analysis was an inclusion criterion for the systematic search, resulting in all 84 (100%) studies incorporating at least one spectroscopic analysis. ATR-FTIR was the most widely used technique for evaluating chemical changes in microplastics, as reported in 74 articles (88.1%). Other spectroscopic techniques applied were XPS, UV–Vis, Raman, u-Raman, u-FTIR, and AFM-IR. Vibrational spectroscopy is the most established in the field of microplastics. Fourier transform infrared spectroscopy (FTIR) and Raman spectroscopy are employed worldwide, including in Latin America (Fernandes et al. 2022), for the chemical identification in environmental samples and for evaluate the degradation levels of these polymers through the emergence and increase in intensity of carbonyl (= O) and hydroxyl bands (O–H) (Cacuro et al. 2018). The microscopy-coupled techniques (μ-FTIR and μ-Raman) are less frequently used due to their inherent limitations when compared to ATR-FTIR and Raman spectroscopy. The size and surface roughness, greater radiation scattering, and an increased operational complexity can compromise spectral quality (Chen et al. 2015; Xie et al. 2024). Consequently, the particle sizes analyzed in aging, digestion, degradation, and adsorption/desorption studies included in this review were, in most cases, larger than 50 μm.

**Fig. 7:**
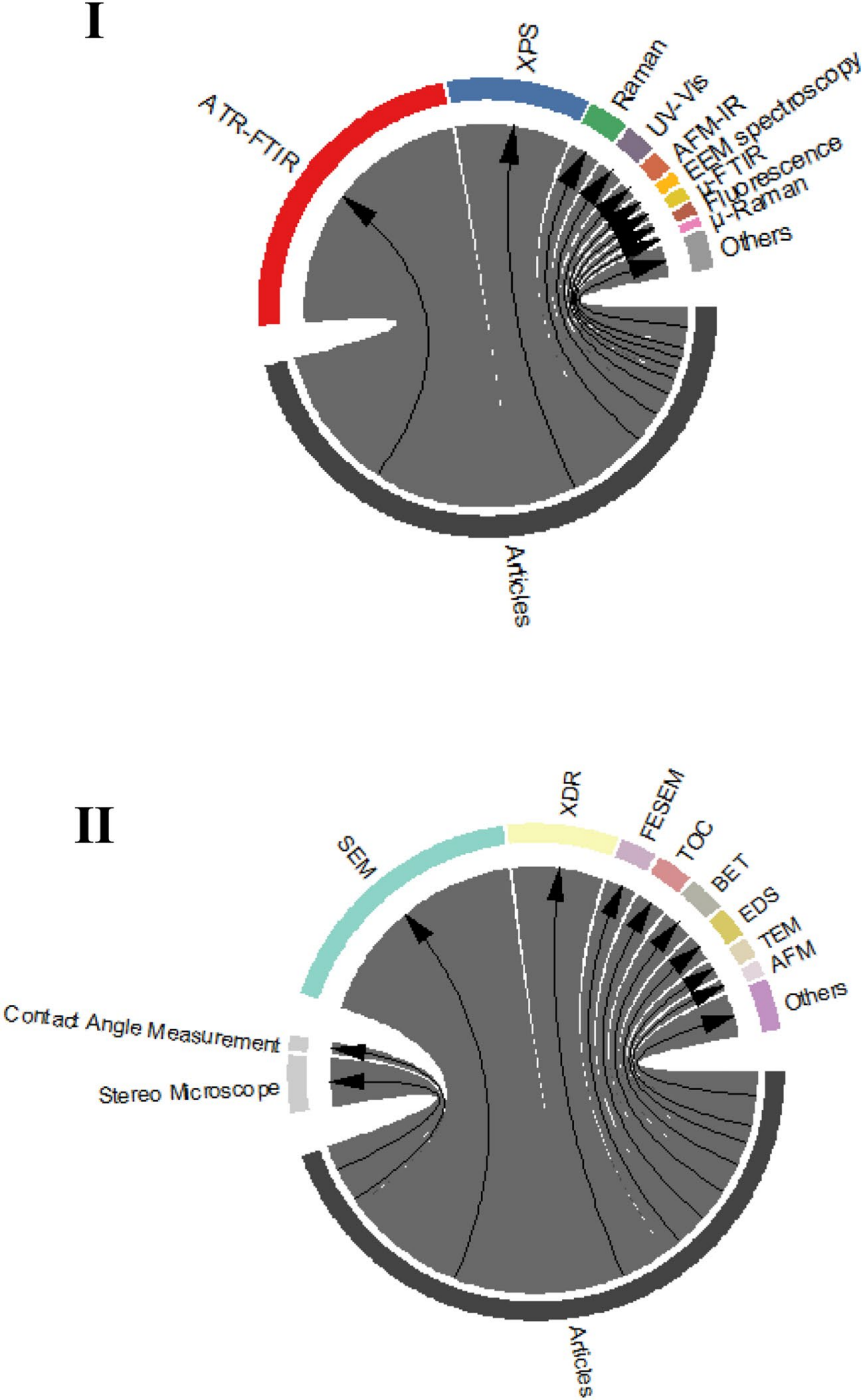
Chemical (I) and physical (II) techniques apply in the characterization of microplastics. **TOC**, total organic carbon analysis; **FESEM**, field emission scanning electron microscopy; **FTIR**, Fourier transform infrared spectroscopy; **Raman**, Raman spectroscopy; **SEM**, scanning electron microscopy; **SEM–EDS**, scanning electron microscopy coupled with energy-dispersive spectroscopy; **XPS**, X-ray photoelectron spectroscopy; **BET**, Brunauer–Emmett–Teller surface area analysis; **UV–Vis**, ultraviolet–visible spectroscopy; **XRD**, X-ray diffraction; **GC–MS**, gas chromatography–mass spectrometry; **DSC**, differential scanning calorimetry; **EEMs**, fluorescence excitation–emission matrices; **TGA**; **EDS**, energy-dispersive spectroscopy; **TEM**, transmission electron microscopy; **AFM**, atomic force microscopy; **μ-FTIR**, micro–Fourier transform infrared spectroscopy; **μ-Raman**, micro-Raman spectroscopy; **AFM-IR**, atomic force microscopy–infrared spectroscopy

The second most frequently reported spectroscopic technique in this review was X-ray photoelectron spectroscopy (XPS), reported in 32 articles (38.0%). XPS can determine the elemental composition and the electronic states at the polymer surface, making it a valuable tool for identifying microplastic particles. The ability to detect functional groups and variations in surface oxygen content makes the technique particularly important for assessing polymer aging. This high sensitivity of XPS to surface-level chemical changes is crucial for elucidating adsorption and desorption processes. However, a major limitation of XPS is the restriction to surface analysis, preventing access to information from deeper layers of the material. For this reason, complementary techniques such as FTIR and Raman spectroscopy are required to obtain a more comprehensive understanding of polymer degradation (Przygoda-Kuś et al. 2025). The study by Di Giulio et al. (2024) demonstrated the effectiveness of XPS by identifying and quantifying chemical groups associated with hydrolysis and oxidation reactions in degraded plastics. Their findings stand out for the synergistic value of combining XPS with FTIR and Raman. While FTIR and Raman spectra typically show only subtle changes in polymers aged over different periods, XPS uniquely reveals chemical modifications occurring at the polymer surface, an area of particular importance, as it constitutes the interface directly exposed to environmental conditions and degradation agents. Figure 7 provides a graphical overview of the chemical and physical techniques used to characterize microplastics.

The scanning electron microscope (SEM) is the predominant technique used for physical characterization, accounting for 62.0% (22 articles) of the selected studies. Stereo microscope, field-emission scanning electron microscope (FESEM), and X-ray diffraction (XRD) were used in 13.1%, 8.3%, and 25% of the articles, respectively. Among these methodologies, SEM is the primary tool for characterizing MPs. Imaging approaches focus on the morphological features of MPs, enabling the identification of geometric configurations and the quantification of surface roughness and smoothness indices, culminating in a comprehensive morphological assessment (Ocampo et al. 2024). High-resolution imaging techniques also elucidate the genesis of nano plastic debris and provide insights into the agglomeration states and dimensional attributes of MPs.

Additionally, XRD analyses provide information on structural stability and properties, including crystal structure, crystallite size, and strain. These contribute to an understanding of MPs behavior across various environmental contexts and their interaction with a diverse number of factors (Ompala et al. 2024; Przygoda-Kuś et al. 2025). This assemblage of information is highly relevant to understanding the dynamics of these materials throughout their aging process and their interactions with various physical and chemical phenomena.

## Aging

Plastics exposed to atmospheric conditions undergo aging attributed to mechanical wear and/or direct exposure to UVC radiation. This phenomenon encompasses alterations in its morphological architecture, hydrophobicity, oxygen concentration, and molecular mass (Liu et al. 2020; Luo et al. 2021). Photoaging is recognized as one of the predominant aging phenomena. The absorption of ultraviolet radiation by established molecular bonds facilitates the generation of radicals that can promote oxidative reactions, in which hydrogen bonds are cleaved, allowing the incorporation of oxygen (Luo et al. 2021).

In ecological systems, MPs coexist with a diverse array of reactive species, including reactive oxygen species generated by natural processes. Nonetheless, there remains a significant gap in understanding the influence of these radicals on the aging dynamics of microplastics, particularly to the extended duration of these processes in natural environments (Liu et al. 2021; Luo et al. 2021). This scenario complicates the entire comprehension of the mechanisms driving the aging of microplastics in natural ecosystems. Consequently, it’s imperative to conduct targeted research on these interactions to effectively evaluate the impacts of oxidant radicals on the aging and behavior of microplastics over time (Liu et al. 2019).

In AOPs applications, aging studies are reported in 41.6% (35 articles) of the research in this review. The aging mechanisms identified can be categorized into two groups: one that investigates hydroxyl (•OH) and sulfate (•SO_4_^−^) oxidizing radicals utilizing agents such as Fenton, persulfate (K_2_S_2_O_8_), ozone (O_3_), and hydrogen peroxide (H_2_O_2_). The second group examines photoaging via UV radiation absorption, along with various combinations of H_2_O_2_, O_3_, NaCO_3,_ and others.

The study by Dong et al. (2022) aimed to clarify the roles of direct (LED lamp, UV, 365 nm) and indirect photolysis in the short- to long-term aging of PS microplastics, using high concentrations of H_2_O_2_ to simulate and accelerate aging. The increase in time and concentration of H_2_O_2_ led to progressive yellowing and dark discoloration of the MPs, indicating a degradation process. Electron microscopy revealed the formation of surface voids and cracks on microplastics during oxidation, leading to fragmentation and increased hydrophilicity. A similar result was found for the study of PS aging accelerated by the UV/H_2_O_2_ oxidative process, which was more significant than the results of tests with UV/H_2_O and UV/Cl_2_ (Liu et al. 2021). The results of direct PS photolysis are similar to those of indirect photolysis, but with lower intensity. Direct photolysis, therefore, is important for aging with low H_2_O_2_ concentrations. FTIR and XPS analysis also confirmed the introduction of oxygenated functional groups on the surface of microplastics during aging. In addition, treatment with visible light/H_2_O_2_ showed less significant results than UV/H_2_O_2_ due to the difficulty in decomposing H_2_O_2_ under visible light (Dong et al. 2022). That said, direct photolysis is slow but effective at promoting polymer degradation. However, when combined with oxidizing agents such as H₂O₂ (Kong et al. 2024; Surendran et al. 2025; Wang et al. 2024a), O₃ (Amelia et al. 2022; Du et al. 2023), Na₂S₂O₈ (Ouyang et al. 2022), and Cl₂ (Lee et al. 2025b), the process is accelerated through the generation of reactive species involved in indirect photolysis. This mechanism is environmentally relevant, as polymers in natural settings are often surrounded by these compounds that, once activated, can produce similar radicals and thereby contribute to their aging.

The study by Wang et al. (2023a) investigated another important point: the interaction between MPs and dissolved organic matter in aquatic environments and its impact on the photodegradation of PS. The results obtained show that in the absence of humic acid (HA), the generation of • OH during photodegradation of PS was lower (0.204 mmol L^−1^) than with the presence of HA (0.631 mmol L^−1^) after 40 days of UV irradiation, evidencing the promotion of the formation of • OH from the reduction of O_2_ catalyzed by HA. The results showed a loss of mass, a decrease in particle size, and an increase in oxygenated functional groups. Similar results were reported by Luo et al. (2024), who observed an increase in surface roughness in both pristine and aged PS following the addition of fulvic acid (FA). Infrared and Raman spectroscopy analyses indicated stronger interactions between MPs and FA in aged samples, accompanied by a higher abundance of oxygen-containing functional groups on the particle surfaces. In saline environments, changes in the surface morphology, roughness, size, and hydrophobicity of PS are further intensified. The increase in salinity has been shown to cause surface cracking, with fissures becoming deeper as salinity levels rise (Zhang et al. 2025). These results indicate that the organic matter present in aquatic systems accelerates the photodegradation of MPs, modulates their fragmentation and adsorption, and influences their fate in aquatic ecosystems.

The second group verifies the effects of chemical oxidants that generate powerful radicals, simulating the accelerated aging of polymers. Liu et al. (2019) investigated the impact of AOPs treatments on polystyrene (PS) and polyethylene (PE) microplastics in simulated aquatic environments. After 10 and 30 days of treatment, both polymers showed surface cracks, with the heat-activated K_2_S_2_O_8_ system demonstrating greater oxidizing capacity than either th O_3_ and or Fenton systems. Luo et al. (2021) also found similar results when evaluating the effects of O_3_, Fenton, and K_2_S_2_O_8_ treatments on the degradation of low-density polyethylene (LDPE) microplastics. Among the three oxidants, persulfate has been shown to induce more pronounced cracks on the surface of microplastics, with an average size of 206 nm. A considerable increase in PVC crystallinity was also the result obtained after treatment with persulfate activated by UV radiation (Ouyang et al. 2022), heating for PLA, PP, PE, PS and PET (Kong et al. 2024; Li et al. 2024b; Zhang et al. 2023a), and Fe^2+^ for PE (Dai et al. 2024) particles. The studies demonstrate that chemical oxidants can accelerate the aging process, and activated persulfate is the most efficient under near ambient conditions. The likely reason for this efficiency is the ability to generate •SO_4_, which, among the radicals, is the greatest in oxidizing power.

The chemical changes induced by the treatments also affected the surface properties of the microplastics. The hydrophobicity of microplastics decreased after PE treatment, showing greater hydrophilicity than PS due to the formation of hydrophilic groups. The structural properties of microplastics were also modified by the treatments, resulting in new signals at 1691, 1706, 1712, 1722, 1732, and 1770 cm-1 for PS, and at 1712, 1719, 1735, and 1780 cm-1 for PE in the FTIR spectra. The different peak intensities also suggest distinct aging mechanisms among the studied polymers. In addition, the acceleration of natural alteration processes was evidenced, and the treatment with K_2_S_2_O_8_ was more efficient in this regard. For comparison, an increase in the carbonyl signal, albeit to a lesser extent, was also observed in groups treated with Fenton, consistent with the morphology analysis (Liu et al. 2019). The average carbonyl index generated by the Fenton process was also significantly higher than that from UV-induced aging, suggesting a more substantial impact on the aging of these particles in aquatic environments (Liu et al. 2023). The complexity of the interactions between microplastics and oxidizing agents is apparent. It depends on the relationship between the radicals produced and the types of microplastics because they follow distinct aging mechanisms. All that provides valuable information for understanding the behavior and environmental impact of these pollutants. Table 1 presents a compilation of references from the review that address the application of AOPs for the aging of microplastics.

**Table 1:** Reference table of AOPs applications for aging. **FT-ICR-MS**, Fourier transform ion cyclotron resonance mass spectrometry; **3D-EEMs**, three-dimensional fluorescence excitation–emission matrices; **TOF–MS**, time-of-flight mass spectrometry; **DSA**, drop shape analysis; **CA**, contact angle measurement; **GC–MS**, gas chromatography–mass spectrometry; **DSC**, differential scanning calorimetry; **TGA**, thermogravimetric analysis

Microplastics	AOPs	Characterizations	References
PS e HDPE,	K_2_S_2_O_8_, Fenton,	FESEM and FTIR;	(Liu et al. 2019)
HDPE, PP e PVC	Fenton,	FTIR and XPS	(Liu et al. 2023)
PS	H_2_O_2_/UV	Stereomicroscope and FTIR	(Liu et al. 2021)
PS, PA6	Fenton, PMS	FESEM, SEM, FTIR, XPS and Raman	(Liu et al. 2022a)
PS	H_2_O_2_/UV, UV	FESEM, FTIR and XPS	(Dong et al. 2022)
PS, HDPE, PP, PCL	H_2_O_2_/HNO3	Stereomicroscope and ATR-FTIR	(Zhang et al. 2023b)
LDPE	Na_2_S_2_O_8_, Fenton, O3	SEM and AFM-IR	(Luo et al. 2021)
PVC	Na_2_S_2_O_8_/UV	SEM and AFM-IR	(Ouyang et al. 2022)
PVC	UV/Ác.O e Fe(III)	BET, SEM, µ-FTIR and Raman	(Wang et al. 2020)
PP, PET E PA6	H_2_O_2_/UV, O_3_/UV e UV	SEM, FTIR and EEMs	(Du et al. 2023)
PP	UV	FESEM, SEM and ATR-FTIR	(Wu et al. 2021)
PS	UV/Na_2_CO_3_	XDR, SEM, ATR-FTIR and XPS	(Wang et al. 2024b)
PS	UV	SEM, XPS, FTIR and CA	(Wen et al. 2023)
PP, PET e PLA	K_2_S_2_O_8_	SEM, ATR-FTIR, BET and UV–VIS	(Zhang et al. 2023a)
PE	O3 e O3/H2O2	ATR-FTIR	(Amelia et al. 2022)
PS	UV	EDS, ATR-FTIR, XPS	(Wang et al. 2023c)
PS and PLA	K_2_S_2_O_8_/Heat	SEM, ATR-FTIR, XPS and XDR	(Cheng et al. 2024)
PE	K_2_S_2_O_8_/Fe^2+^	SEM–EDS, FTIR and XPS	(Dai et al. 2024)
PE, PP and PS	K_2_S_2_O_8_/Heat, UV/H_2_O_2_ and UV-PAA	BET, FTIR, SEM, XPS, XDR and DSA	(Kong et al. 2024)
PLA	UV	FTIR, UV–Vis	(Li et al. 2024a)
PP	K_2_S_2_O_8_/Heat	SEM, FTIR, XPS and Raman	(Li et al. 2024b)
PS	UV	SEM, XPS, FTIR, Raman and AFM-IR	(Luo et al. 2024)
PE	KMnO_4_/UV	FESEM, FTIR, XDR, UV–Vis, XPS, Raman, TGA, DSC, TOC, and 3D-EEM	(Nguyen et al. 2024)
PE, PS and PET	K_2_S_2_O_8_/Fe^2+^	SEM and ATR-FTIR	(Ning et al. 2024)
PLA	UV	SEM, FTIR, XPS and XDR	(Sun et al. 2024a)
PVC	UV/SPC	FTIR, SEM, XDR, XPD, BET and GC–MS	(Su et al. 2024)
PE, PP, PS and PVC	UV/H_2_O_2_	SEM, XPS and FTIR	(Wang et al. 2024a)
PS, PET and PVC	UV/Cl 2 and UV/NH 2 Cl	FT-ICR-MS, SEM, FTIR, TOC, and EEMs	(Zheng et al. 2024)
PLA	UV	SEM–EDS, BET, XDR, FTIR and XPS	(Feng et al. 2025a)
PE and PLA	UV, Cl_2_ and UV/Cl_2_	FTIR, TOC-CHP, EEM, and FESEM	(Lee et al. 2025b)
PVC, PS and PET	H_2_O_2_	SEM, FTIR and XPS	(Li et al. 2025)
PE	UV	FTIR	(Mostefaoui et al. 2025)
PS, PP, PET and PLA	Fe^2+^/PMS	SEM, CA, FTIR, XPS, XDR and BET	(Feng et al. 2025b)
PLA	UV	SEM, FTIR, XPS and XDR	(Sun et al. 2025)
EPDM	UV and UV/H_2_O_2_	FTIR	(Surendran et al. 2025)
PVC	UV	SEM, FTIR, XPS, DSA, 3D-EEM and XDR	(Tumrani et al. 2025)
PS	UV	SEM, FTIR, UV–Vis, EEM, TOF and XDR	(Zhang et al. 2025)
PLA	UV/Fenton	SEM, FTIR and (μ-FT-IR	(Zhong et al. 2025)

## Microplastic degradation

Plastic particles are considered emerging pollutants due to their widespread occurrence in industrial discharges and urban wastewater (Zhou et al. 2022). Conventional wastewater treatment processes do not completely retain either primary or secondary microplastics. Consequently, these particles are often carried through channels and rivers into freshwater and oceanic ecosystems (Belé et al. 2021). Coagulation, sedimentation, and filtration in wastewater treatment can reduce microplastic levels by 5–95% depending on their dimensions, shapes, and polymer types in specific scenarios, but remain a challenge (Chen et al. 2022; Ortiz et al. 2022). The breakdown of polymers, even those labeled as biodegradable, involves processes that take a considerable amount of time to decompose in nature. However, AOPs approaches are acknowledged as a promising method for degrading plastic waste. Oxidation facilitated by reactive radicals leads to the fragmentation of the polymer chain, the formation of hydroxyl and carbonyl functional groups in MPs, and a decrease in molar mass, ultimately enabling complete mineralization (Amelia et al. 2022).

MPs degradation accounts for 26.2% (22 articles) of the AOPs applications identified in the bibliographic analysis. The Fenton oxidation technique is considered an attractive approach due to its high cost-effectiveness and applicability in MPs research, and it is consistently reported in the degradation studies. That said, Ortiz et al. (2022) investigated the fate of MP during Fenton oxidation, using different types of plastics (PET, PP, PS, and PVC). Results demonstrated that the mass loss was low, approximately 10%, despite a reduction in the size of MPs particles after their mineralization. The study using photo-Fenton and Fenton-like system based on a TiO_2_/graphite cathode process was also unsuccessful, indicating the need to explore alternative strategies (Bule Možar et al. 2024). A similar mass loss ($\mathrm{10,23}\mp \pm \mathrm{1,91} \%$) from PET has also been reported in the literature using a heterogeneous AOPs (Bi_2_O_3_@N-TiO_2_) with TiO_2_ as the photocatalyst. However, when performed without the hydrolysis of the basic medium, the result was even lower ($\mathrm{3,27}\pm 1 \%$) (Zhou et al. 2022). Other heterogeneous strategies were also tested, such as piezo-photocatalysis (Lu et al. 2025; Wu et al. 2025) and electron-Fenton (Wang et al. 2025). The degradation rates were moderate but still relatively low (28.90–58.46%) in terms of mass reduction for PS and PET, with the electron-Fenton process showing comparatively better performance.

Considering the limitations of Fenton-based processes, recent studies have explored persulfate activation as an alternative. Persulfate is widely used to degrade persistent pollutants; its excellent water solubility and low production costs are important attributes for a homogeneous oxidation process (Dai et al. 2024). The oxidant can be activated to produce reactive radicals through various methods, some ecological and others requiring some energy input. Easton et al. (2024) tested light, heat, and ultrasound to activate persulfate at different temperatures and concentrations to degrade polyester fiber. Optimized UVC/persulfate conditions (31.8 mW cm^−2^, 500 mg L^−1^) resulted in an 18.5% mass loss over 9 h, with clear surface pitting and cracking. Heat alone was considered a poor activator, while ultrasound produced a 16.0% reduction in mass, accompanied by increased fragmentation and the formation of smaller MPs and nanoparticles. The study by Dai et al. (2024) aimed to use Fe^2+^ as a catalyst (S-nZVI@ATP) for heterogeneous oxidation to degrade PE under different conditions. After 24 h, the average size of PE decreased from 64.2 ± 0.86 μm to 28.1 ± 0.91 μm (S/Fe = 0.75:1, catalyst dosage of 0.3g, pH = 6, temperature = 35 • C, and persulfate concentration = 20 mmol/L-1), corresponding to a degradation efficiency of 43.80%.

In this context, despite low to moderate mineralization efficiencies across different traditional Fenton and Persulfate-based processes, the particles exhibited significant physicochemical changes. These include the formation of wrinkles, voids, and surface pits, as well as chemical modifications marked by the incorporation of oxygen containing functional groups, which increase particle hydrophilicity (Dai et al. 2024; Easton et al. 2024; Ortiz et al. 2022). Furthermore, they can form degradation products (Thirunavukkarasu et al. 2024). Overall, the results demonstrate how these materials are resistant to degradation. Traditional AOPs can cause significant physicochemical transformations, but their mineralization capacity is still limited. So, developing more efficient methods, such as hydrothermal and electron-Fenton processes, is necessary to mineralize these contaminants.

To address these gaps in mineralization efficiency, Hu et al. (2022) explored combining elevated temperatures in a hydrothermal process with the Fenton reaction. Analysis of the chemical, structural, and morphological properties of MPs (PS, HDPE, LDPE, PP, PET, and PA6) in water revealed a significant influence of hydrothermal conditions on polymer removal. The approach successfully eliminated 95.9% of the MPs mass during the period from the 4th to the 16th hour of the study. The investigations showed that ·OH radicals are primarily responsible for degradation. The most effective conditions were strongly acidic pH (0.7–1.0), high temperatures (140–160 °C), and elevated concentrations of Fe^2^⁺ and H₂O₂.

Other combinations found to have better mineralization efficiency included combining electricity with Fenton and Persulfate in electro-Fenton activation systems. Simultaneously, electrolysis (Eqs. 6 and 7) is used to activate Fenton (Eq. 8) and persulfate (Eq. 9 and 10) reactions by generating H₂O₂ and releasing electrons, thereby promoting the formation of large quantities of highly oxidizing radicals (Sun et al. 2024b). The degradation of PE in a heterogeneous electro-Fenton-activated persulfate system under optimized conditions (60 mg/L PE, 40 mM persulfate, 150 mg Fe_3_O_4_, 20 h treatment) reached 90.6% degradation (Gao et al. 2025). Comparable performance was observed for PET, with 91.3 ± 0.9% removal of 100 mg/L after 12 h, and in that case, even nanoplastics formed during the process were degraded at specific points (Lin et al. 2024). So, while these methods are exceptionally effective in aqueous environments, there is a need to create systems that can be applied on a larger scale, minimizing the usage of reagents and energy. The application of these techniques to samples rich in organic matter, such as sludge and industrial wastewater, is another area that should be explored.

6 $${O}_{2}+2{H}^{+}+2{e}^{-}\to {H}_{2}{O}_{2}$$

7 $${Fe}^{3+}+{e}^{-}\to {Fe}^{2+}$$

8 $${Fe}^{2+}+{H}_{2}{O}_{2} \to {Fe}^{3+}+ \cdot OH+ {OH}^{-}$$

9 $${S}_{2}{O}_{8}^{2-}+{e}^{-}\to {SO}_{4}^{2-}+{ \cdot SO}_{4}^{-}$$

10 $${Fe}^{2+}+{S}_{2}{O}_{8}^{2-}\to {Fe}^{3+}+{\cdot SO}_{4}^{-}+{SO}_{4}^{2-}$$

Wet Oxidation with Air (WAO) is a process of oxidation applied to manage highly concentrated, toxic, and dangerous effluents. Organic contaminants are oxidized and broken down into CO_2_ and H_2_O using oxygen or air as the oxidizer at elevated temperatures (125–320 ℃) and pressures (0.5–20 MPa) (Zhang 2020). Over the years, the wet oxidation technique has advanced rapidly, broadening its use as a substitute for MPs removal in effluents (Wang et al. 2023a; Wang et al. 2023b). The technology is currently under research and remains in the experimental stage due to the substantial initial investment required for the reaction apparatus. The WAO method comprises four phases: induction, proliferation, degradation, and the final phase. In the induction and proliferation phases, molecular oxygen is thought to play a role in generating various radicals, such as ·HO, ·RO, ·ROO, which target organic substances, aiming to oxidize them into less harmful inorganic materials (Zhang 2020).

In the research conducted by Wang et al. (2023a), the breakdown of PE, PS, and PET microplastics was examined under varying temperatures (200, 220, 240, and 260ºC) and oxidation scenarios. Pure water and microplastics were used to create synthetic samples for studying microplastic degradation without the influence of other substances. The dissolved organic carbon (DOC) levels in the microplastic flows exhibited a steady rise or an initial sharp increase followed by a gradual decline within 120 min, influenced by the reaction temperatures and types of microplastics. In general, elevated oxygen pressure led to faster decomposition and breakdown of the MPs, with hydroxyl radicals formed during wet air oxidation (WAO) being key to the oxidation and degradation of the materials. In this case, temperature was an important variable in accelerating the reactions and in relation to the decomposition of DOC. The findings indicated a mineralization of 66% for PE mass and 84.5% for PS.

In comparison, 77.3% of the PET mass was retained in the solid state, with only 20.4% dissolved and oxidized into low-molecular-weight soluble compounds. WAO experiments aimed at evaluating the concurrent degradation of microplastics and sludge were conducted, revealing that some organic matter in the sludge was oxidized or mineralized, with no particulate matter detected in the solid residues (Wang et al. 2023b). The results of this type of treatment for small particles of MPs (<  50 mm) and nanoplastics in industrial waste samples were also very fruitful and effective, with mineralization rates of 94.8–98.6% (Hu et al. 2024). Together, this evidence makes WAO a promising technology for the advanced removal and mineralization of micro- and nanoplastics, with particular effectiveness in complex matrices such as high-organic-load and industrial wastewater. Table 2 provides references regarding the use of AOPs for the degradation of microplastics.

**Table 2:** Reference table of AOPs applications for degradation. **PES**, polyester; **AFM**, atomic force microscopy; **CHNS**, elemental analysis by combustion; **FEG-SEM**, field emission gun scanning electron microscopy; **LC–MS**, liquid chromatography–mass spectrometry; **μ-FTIR**, micro–Fourier transform infrared spectroscopy; **py-GC–MS**, pyrolysis–gas chromatography–mass spectrometry; **LC-QTOF-MS**, liquid chromatography–quadrupole time-of-flight–mass spectrometry; **EDS**, energy-dispersive spectroscopy; **TGA–DSC/MS**, thermogravimetric analysis–differential scanning calorimetry/mass spectrometry; **HPLC-HRMS**, high-performance liquid chromatography–high-resolution mass spectrometry; **ICP-OES**, inductively coupled plasma optical emission spectrometry; **TEM**, transmission electron microscopy

Microplastics	AOPs	Characterizations	References
PE, PP and PET	Bi_2_O_3_@N-TiO_2_	XDR, SEM, ATR-FTIR	(Zhou et al. 2022)
PS, HDPE, LDPE, PP, PET and PA6	Fenton	SEM, FTIR, XPS and Raman	(Hu et al. 2022)
PS, PE, PP, PET and PVC	Fenton e UV	Stereomicroscope, TOC, CHNS, SEM and ATR-FTIR	(Ortiz et al. 2022)
PS, PE, PET	O_2_ (WAO)	ATR- FTIR, TOC, FEGSEM, Raman and XPS	(Wang et al. 2023b)
PET	H_2_O_2_/UV	Microscópio óptico, SEM,—ATR-FTIR	(Easton et al. 2023)
PS, PE, PET	O_2_ (WAO)	ATR- FTIR, TOC, FEGSEM, Raman and XPS	(Wang et al. 2023a)
PE	Na_2_S_2_O_8_/Fe^2+^	LC–MS, TOC, SEM–EDS, FTIR, XDR and XPS	(Dai et al. 2024)
PES	Na_2_S_2_O_8_/Light, heat and ultrasound	SEM, AFM, ATR-FTIR and UV–Vis	(Easton et al. 2024)
PS, PMMA, PE, PP, PET, PLA, PBAT and PVC	O_2_ (WAO)	μ-FTIR and py-GCMS	(Hu et al. 2024)
PET	Na_2_S_2_O_8_/electro-Fenton-activated	SEM, XDR, XPS, ATR-FTIR, TOC, UV–Vis, LC-QTOF-MS	(Lin et al. 2024)
LDPE, PP and PVC	Photo-Fenton and Fenton	ATR-FTIR and SEM	(Bule Možar et al. 2024)
PVC	Na_2_S_2_O_8_/UV- and electricity	SEM, EDS, XRD, ATR-FTIR and LC–MS	(Huang et al. 2024)
PE	Mt- Na_2_S_2_O_8_-EF	SEM, FTIR, TOC and XPS	(Gao et al. 2025)
PS	Photo-Fenton	ATR-FTIR, TOC, TGA–DSC/MS, NMR and HPLC-HRMS	(Thirunavukkarasu et al. 2024)
PS	Piezo-photocatalytic	SEM, FTIR, EPR and LC–MS	(Lu et al. 2025)
MP and Midrofibers	SnO_2_-Nb_2_O_5_	FTIR	(Nhan et al. 2025)
PE	Na_2_S_2_O_8_/ZnO	FTIR, SEM and TOC	(Sharara et al. 2025)
PE	O_3_	ATR-FTIR and TOC	(Topkaya et al. 2025a)
MP	O_3_	FTIR	(Topkaya et al. 2025b)
PVC	Electro-Fenton	XDR, SEM, XPS, FTIR, UV–Vis, TOC, LC–MS and ICP-OES	(Wang et al. 2025)
PET	Piezo-photocatalytic	SEM, TEM, XDR, XPS,FTIR and UV–Vis	(Wu et al. 2025)

## Digestion

Environmental samples such as water, sediment, and biota exhibit varied amounts and qualities of organic matter influenced by environmental factors (Alfonso et al. 2021; Enders et al. 2017). A range of digestion methods for MPs analysis is employed, using alkaline, acid, and oxidant techniques that differ in reagents, concentrations, procedures, and processing times. Nonetheless, recent assessments of the effectiveness of these digestion methods have revealed alterations in the structural and chemical properties of MPs, reflected in changes in color, weight, and structure of the polymers across different digestion protocols (Alfonso et al. 2021). These underscore the importance of selecting appropriate digestion methods tailored to specific environmental samples, as they can significantly affect the precision and reliability of microplastic characterization.

Among the different methods, the use of oxidizing agents has proven a viable alternative for digesting various sample matrices. In this review, applications of AOPs for the disintegration of organic matter account for 21.43% (18 articles). Fenton and H_2_O_2_ are the most common oxidizing agents reported in the literature for environmental samples (Arthur et al. 2009). They are suggested to avoid damage to polymers caused by acid- or alkaline-based methods (Gao et al. 2024). That sad, the development or improvement of existing protocols has importance to this field of research.

Pfeiffer and Fischer (2020) evaluated Fenton, H_2_O_2_, and NaClO at different concentrations and temperatures for water and sediment samples. In efficiency assessments, three types of organic matter were scrutinized: soft plant tissue (leaves), hard plant tissue (branches), and calcareous shells (mussels). The results indicated that H_2_O_2_ demonstrated the highest efficiency in breaking down organic matter in soft (71.1%) and complex (58.9%) tissues, especially with concentration increases from 30 to 50% and temperature elevations from 20 to 60–70°C. The Fenton reagent also yielded similar results in the digestion of soft and hard tissues, achieving up to a 64.4% reduction in organic matter mass. In contrast, the application of NaClO produced even better results compared to H_2_O_2_, leading to mass reductions of up to 88.0% for soft tissues and 92.1% for hard tissues.

Comparable degradation outcomes were observed in soil samples, with degradation rates ranging from 75–87% for H_2_O_2_ (30%) at 45ºC and for Fenton at room temperature (Tan et al. 2022). The consistency in findings at lower temperatures may be linked to the differing compositions of organic matter studied in the research. The exposure of the examined polymers (PS, HDPE, LDPE, PP, and PET) to the oxidants H_2_O_2_ and NaClO had negligible effects, except for PA6, which showed an 8.7% mass loss when subjected to elevated temperatures (60—70 ºC) during the digestion processes (Tan et al. 2022). Consequently, NaClO emerged as the oxidant that produced the most favorable outcomes regarding organic matter degradation while having the least effect on microplastics. These results confirm that oxidant-based digestion methods can effectively remove organic matter while essentially preserving the integrity of most microplastic polymers. However, standardizing these methods remains a challenge, especially given the large number of environmental matrices that are contaminated.

In the literature, various protocols for the digestion of organic matter in different environmental samples are reported (Table 3). Huang et al. (2025) proposed optimizing digestion conditions (concentration, time, and temperature) and type (Alkaline, Acid, and Oxidative) across 27 digestion protocols. The efficiency of removing organic matter was evaluated from four typical samples (animal tissue, plants, soils, and sludges) and 26 types of MPs. The results show that for animal tissue, that 2% KOH at 40 ◦C were the most efficient condition and was responsible for a removal rate of 97.5%, however, other studies have shown that persulfate in a basic medium (NaOH) can also provide rapid digestion with minimal MPs degradation (Gao et al. 2024). On the other hand, Fenton’s reagent at 60 ◦C for plants reached 94.1%−97,7% of removal of organic matter depending on the temperature used. To the soil sample, both 0.2 M K₂S₂O₈ at 60 ◦C and Fenton have the highest rates, 13.1% and 13.3%, respectively. But Tan et al. (2022) reported higher Fenton results for this type of sample, even at lower temperatures, suggesting that sample composition may have affected the results. Sludge samples are complex in relation to organic matter. After several tests, a three-step protocol was necessary (0.2 M K₂S₂O₈ at 65 ◦C, 15% H₂O₂ at 40 ◦C, and 2% KOH at 40 ◦C) with a removal rate of 63.6%. These demonstrate that the choice of digestion method depends heavily on the matrix being analyzed, and that optimized protocols are essential to balance high efficiency in removing organic matter with minimal degradation of MPs. Methodological studies are crucial before initiating monitoring of this pollutant to obtain reliable results.

**Table 3:** References for the use of AOPs for the digestion of organic matter in environmental samples. **EPS**, expanded polystyrene; **ABS**, acrylonitrile–butadiene–styrene; **PU**, polyurethane; **AS**, acrylonitrile–styrene; **EVA**, ethylene–vinyl acetate; **COC**, cyclic olefin copolymer; **COP**, cyclic olefin polymer; **PBS**, poly(butylene succinate); **PCL**, poly(ε-caprolactone); **PETG**, polyethylene terephthalate glycol-modified; **PHA**, polyhydroxyalkanoates; **POE**, polyolefin elastomer; **POM**, polyoxymethylene; **PPS**, polyphenylene sulfide; **SPS**, syndiotactic polystyrene; **TSB**, thermoplastic styrene block copolymer. **μ-Raman**, micro-Raman spectroscopy; **SDS**, sodium dodecyl sulfate (C₁₂H₂₅NaSO₄)

Microplastics	AOPs	Characterizations	Matrix	References
PS, EPS, PC, HDPE and LDPE	H_2_O_2_	SEM and ATR-FTIR	Water	(Alfonso et al. 2021)
PS, HDPE, LDPE, PP, PET and PA6	Fenton, H_2_O_2_ e NaClO	µ-Raman	Water and sediment	(Pfeiffer and Fischer 2020)
PS	H_2_O_2_	Stereomicroscope, SEM, and ATR-FTIR	Biological	(Gulizia et al. 2022)
PS, PC, PE, PP, PET, PA6, ABS, PMMA and PVC	NaClO	Raman	Gastrointestinal tract	(Enders et al. 2017)
PS, PE and PMMA	Fenton	Optical microscope and µ-FTIR	Sewage, effluent and sludge	(Cunsolo et al. 2021)
PS, PE, PP, PET, PA6 and PVC	H_2_O_2_,	Stereomicroscope and ATR-FTIR	Sediment	(Duan et al. 2020)
PS, PC, HDPE, LDPE, PP, PET, PA6, PU, PMMA and AS	Fenton and H_2_O_2_	Optical microscope, SEM, ATR-FTIR	Soil	(Tan et al. 2022)
PS, PC, PE, HDPE, LDPE PP, PET and PA6	H_2_O_2_/Enzyme, H_2_O_2_/HNO_3_	Stereomicroscope and ATR-FTIR	Exoesqueleto	(Kallenbach et al. 2021)
PA, PE, PP, PET and PS	H_2_O_2_ and C_12_H_25_NaSO_4_	Raman and Stereomicroscope	Planktonic biomass	(Tuuri et al. 2024)
PC, PA6, PBT, PS, PVC, PP, PE and EVA	K_2_S_2_O_8_/NaOH,,Fenton and H_2_O_2_	SEM–EDS and FTIR	Biological tissues	(Gao et al. 2024)
ABS, AS, COC, COP, EVA, HDPE, LEPE, PA66, PBAT PBS,, PBT, PC, PCL, PET, PETG, PHA, PLA, PMMA, POE, POM, PP, PPS, PS, PVC and SPS	H_2_O_2_, Fenton and K_2_S_2_O_8_	DSA, Stereomicroscope, μ-Raman and ATR-FTIR	Animal tissue plant tissue, soil and sewage sludge	(Huang et al. 2025)
PE, PP, PVC, TSB, and PMMA	K_2_S_2_O_8_/Heat-NaOH	SEM, EDS, FTIR, Raman and XPS	Natural water	(Lee et al. 2025a)

MPs recovery studies encompass 9 publications on the use of oxidizing agents to break down organic matter, covering samples from effluents, drinking water, soil, soft tissues, sediments, exoskeletons, and aquaculture feed. The predominant AOPs used are Fenton, H_2_O_2_, and H_2_O_2_/HNO_3_, aimed at assessing the effectiveness of digestion protocols and their influence on microplastics, as well as their implications for quantifying environmental samples. Savino et al. (2022) investigated the efficacy of the oxidative digestion method using Fenton at varying temperatures (75, 50, and 30º C), different volumes of H_2_O_2_ (100 or 60 mL of H_2_O_2_ + 20 mL FeSO_4_ ▪ 7H_2_O), and various particle sizes (5 mm — 1 mm; 1 mm — 500 µm; 500 µm—100 µm) to isolate and extract pristine microplastics (PS, PP, PE, PA, PVC, and PET) from a soil matrix. The trials conducted at various temperatures and peroxide volumes showed recovery efficiencies of approximately 100% for the majority of the tested MPs.

The primary differences in efficiency were noted with the PVC and PET samples. The recovery rate for PVC particles exceeded 100% in every experiment, with the rate increasing with the temperature of the tested systems (108–170%). The observed increase in the number of particles relative to the initial count can be attributed to the aggressive nature of the digestion treatment on PVC, which fragmented it into smaller particles observed and identified in the sample. Conversely, the recovery rate for PET fragments was the lowest among all polymers (24—93%). This loss of particles is primarily due to the matrix effect, which traps particles within the background and complicates recovery efforts. Tests involving smaller microplastics showed 100% recovery across all six treatments. However, a similar outcome was not observed for PVC and PET by Duan et al. (2020), who used digestion with 30% H2O2 (70— 100 °C) to recover particles from the sediment matrix, achieving a recovery rate of 100%. This variance may stem from the oxidizing agent's reduced aggressiveness, the matrix type, and the particle size. The two-step treatment (H_2_O_2_ + HNO_3_), as observed with PET, yielded a diminished recovery rate in the recovery study by Zhang et al. (2023b) for PS particles. Digestion with 0.5 M HNO_3_ produced higher recovery efficiencies (100 μm: 86.85%; 200 μm: 94.72%). In contrast, lower recovery rates were observed as the acid concentration increased, as particles may degrade at elevated concentrations, complicating recovery.

A study conducted by Ge et al. (2024) compared three digestion techniques (H_2_O2 30%, Fenton, and H_2_O_2_ 30% + HNO_3_) to evaluate the recovery efficiencies of MPs in aquaculture feed samples. MPs particles (PE, PP, PET, PS, and PS) in two size ranges (20—50 µm and 1—2 mm) were subjected to digestion at varying temperatures (40, 50, and 60º C) over a duration of 48 h. The mean recovery efficiencies recorded were 93.5 ± 1.8%, 88.2 ± 2.3%, and 96.1 ± 1.5%, respectively. For the larger particles (1—2 mm), the average recovery efficiencies for all three digestion methods were around or exceeded 100% (101.0 ± 2.6%, 95.6 ± 4.5%, and 118.4 ± 7.3%), respectively. The increased contact area with the oxidizing agents might have led to the H_2_O_2_ 30% + HNO_3_ treatment producing more particles than initially present for PE, PP, PET, and PS MPs. In conclusion, the findings indicated that digestion with 30% H_2_O_2_ was the most effective method for determining the actual presence of MPs in aquaculture feeds. Collectively, these studies demonstrate that MPs recovery efficiency varies substantially with matrix type, polymer characteristics, particle size, and oxidant strength. While oxidative digestion is broadly effective, overly aggressive conditions can lead to MPs degradation or fragmentation, biasing quantification. Table 4 lists references on the application of AOPs to assess the efficiency of MPs extraction following digestion.

**Table 4:** References for the AOPs application assessing the efficiency of extracting microplastics. **FEG-SEM**, field emission gun scanning electron microscopy

Microplastics	AOPs	Characterizations	Matrix	References
PS, HDPE, PP, PCL	H_2_O_2_/HNO_3_	Stereomicroscope, ATR-FTIR	Wastewater	(Zhang et al. 2023b)
PS, PE, PET e PVC	KMnO_4_	MEV-FEG, FTIR, XPS	Drinking water	(Chen et al. 2022)
PS, PE, PP, PET, PA6 e PVC	Fenton	Stereomicroscope, SEM e ATR-FTIR	Soil	(Savino et al. 2022)
PS, PE, PP, PET, PVC, LDPE e PA	H_2_O_2_	Raman	Bivalve tissue	(Thiele et al. 2019)
PS, PE, PP, PET, PA6 e PVC	H_2_O_2_	Stereomicroscope, ATR-FTIR	Sediment	(Duan et al. 2020)
PS, PE, PP, PET, PVC, e PA	Fenton	μ-FTIR	Wastewater and sludge	(Cunsolo et al. 2021)
PS, PC, PE, HDPE, LDPE PP, PET, PA6,	H_2_O_2_/Enzima, H_2_O_2_/HNO_3_	Stereomicroscope, ATR-FTIR	Exoskeleton	(Kallenbach et al. 2021)
PS, PE, PP e PET,	H_2_O_2_, H_2_O_2_/HNO_3_ e Fenton	LC–MS, SEM e ATR-FTIR	Aquaculture feed	(Ge et al. 2024)
ABS, AS, COC, COP, EVA, HDPE, LEPE, PA66, PBAT, PBS, PBT, PC, PCL, PET, PETG, PHA, PLA, PMMA, POE, POM, PP, PPS, PS, PVC and SPS	H_2_O_2_, Fenton and K_2_S_2_O_8_	DSA, Stereomicroscope, μ-DSA, Stereomicroscope, μ-Raman and ATR-FTIR	Animal tissue plant tissue, soil, and sewage sludge	(Huang et al. 2025)

## Adsorption and desorption

Microplastics are not eliminated during standard or oxidative processes in the sewage treatment plant (STP), and residual particles may undergo chemical alterations on their surfaces, altering their ability to absorb pollutants. Such changes make microplastics act as a barrier to the absorption of organic pollutants, reducing the efficacy of oxidative techniques for breaking down contaminants (Belé et al. 2021). That said, the aging of particle surfaces correlates directly with their ability to take in pollutants. Consequently, understanding the interactions between contaminant sorption and desorption and aged microplastics is crucial for assessing the environmental effects of microplastics (Lang et al. 2020).

The research examining the adsorption potential of MPs accounts for 16.67% (16 articles) of the selected literature in this review. The AOPs used for the aging of these MPs included Fenton, Na_2_S_2_O_8_, O_3_, H_2_O_2_, UV, Cl_2_, and their combinations to assess the adsorption of metals, pharmaceuticals, pesticides, and polycyclic aromatic hydrocarbons (PAHs). Lang et al. (2020) investigated the impact of PS aging through Fenton and H_2_O_2_ on the adsorption of Cd^2+^. The findings were adjusted to first-order (H_2_O_2_) and second-order (Fenton) models, revealing that, with prolonged aging time, the ability of PS to absorb Cd^2+^ (0.5 and 2 ppm) increased for both oxidant treatments. When comparing peak adsorption amounts, the second-order model fits the experimental values more closely, suggesting that Fenton exhibits a stronger aging effect than H_2_O_2_. The dynamics of adsorption exhibited a rate of 70 −80% within the initial 6 h, achieving 90—100% by the end of 24 h, marking the peak saturation level. However, Cd^2+^ (1 mg.L^−1^) adsorption by PLA aged by UV radiation for 21 days was faster, with rates of 94.4–96.0% in the first 1.5 h and 100% within 6 h (Sun et al. 2025). Therefore, it can be concluded that the increased specific surface area of the PS and PLA, along with the increase in the quantity of functional groups and biofilm formation, contribute to Pb^2+^ adsorption, and that the characteristics of each type of MPs, the method, and the aging process employed can increase the adsorption efficiency.

The alterations in the characteristics of PE and PLA following aging with O_3_/UV led to increased Pb^2+^ adsorption, which was the purpose of the study by Liu et al. (2022b). Factors in the environment, including pH, ionic strength, and dissolved organic matter (DOM), were considered to gain a deeper insight into the adsorption behavior of this metal on MPs. The results indicated that increasing the environmental pH also enhanced the adsorption capacity for Pb^2+^, consistent with the authors' previous research on the adsorption of other metals, such as Cd, Cu, Zn, and Ag, on MPs. This happened because at low pH (< 7), Pb predominantly exists as Pb^2+^, and the H + ions in solution compete with and displace Pb^2+^ from available adsorption sites. However, the adsorption capacity of MPs decreased with increasing ionic strength and DOM concentration, suggesting that MPs bind not only to heavy metals but also to other ions and organic compounds, competing for binding sites. The composition of the metal solution also contributes to adsorption, as found by Feng et al. (2025a) in their evaluation of the integration of Pb, Cu, and Cd with PLA aged under UV radiation. The adsorption order was Pb(II) > Cu(II) > Cd(II), and the adsorption capacities increased by 22.95%, 17.31% and 26.76%, respectively. In addition, environmental factors such as pH, ionic strength, and DOM also affect the adsorption behavior, as also demonstrated by the study cited earlier. That said, the idea that environmental factors modulate the interaction between heavy metals and aged microplastic particles is accurate. It should be taken into consideration in future research on other contaminants.

The desorption of metals and the decomposition of the organic matter layer at the MPs surface, with the application of Na_2_S_8_O_8_, activated magnetic biochar with porosity and graphitization (PGMB) in natural water, were the main objectives of Ye et al. (2020). Samples were subjected to different treatments, including individual PGMB, Na₂S₈O₈, and Na₂S₈O₈ activated by PGMB, for 4 h. The successful combination of PGMB/Na₂S₈O₈ significantly improved the removal of Pb attached to the microplastic surface, achieving a removal efficiency of over 60%. PGMB rapidly adsorbed Pb from solution while activating Na₂S₈O₈ to degrade organic matter on the microplastic surface, thereby facilitating Pb detachment. The presence of plasticizers and metals in solution resulting from desorption processes is a concern for water and wastewater treatment plants, as they can contribute to the formation of toxic degradation products. Lee et al. (2025b) investigate the effects of typical pre-oxidation treatments (UV, Cl₂, and UV/Cl₂) on the release and transformation of MPs-DOM from polyethylene (PE) and polylactic acid (PLA), and their contribution to the formation of brominated disinfection byproducts (Br-DBP) during subsequent chlorination. Their results showed a substantial increase in DBP formation by two orders of magnitude after oxidative pretreatment, including brominated species associated with plasticizer desorption. UV oxidation was the dominant precursor across all polymer types. That said, the results reveal emerging risks associated with microplastics in water treatment systems, particularly under oxidative conditions. The release of metals, plasticizers, and MPs derived from organic matter can significantly enhance the formation of toxic by-products, underscoring the need for source control and advanced treatment strategies.

MPs can transport organic pollutants in the environment, and the sorption capacities have gained attention in microplastics research. Polycyclic aromatic hydrocarbons (PAHs) are examples of such contaminants commonly found in aquatic environments, particularly in estuaries, due to anthropogenic sources. Zhang et al. (2020) investigated the sorption capacities of three PAHs and PHEs in polyurethane (PT), polyurea (PU), and urea–formaldehyde resin (UF), which are typically used in the plastic industry. MPs were aged in UV light, in H_2_O_2,_ and in H_2_O_2_ with UV light. The characterization indicates the presence of amide and C–O functional groups, increased surface polarity, reduced crystallinity, a more negative zeta potential, and the introduction of polar groups. The data evaluation revealed rapid initial sorption, followed by a steady approach to equilibrium by day 10. PU exhibited the highest sorption capacity for all substances; PT sorption was slower and required longer to reach equilibrium; and UF had the worst result, probably because of differences in the polymer structure. Sorption behavior was influenced by polymer Tg, functional groups, and hydrophobic/polar interactions. The aging process with UV and H₂O₂, and the presence of salinity, reduces these interferences, increasing the sorption capacity of PT and UF, whereas the opposite effect was observed for PU.

Similar behaviors were reported by Kong et al. (2024) in their study on the adsorption of 2-Nitrofluorene (2-Nflu) into PE, PP, and PS. After aging treatments with UV/H₂O₂, UV/PAA, and persulfate/heat, all polymers exhibited increased adsorption capacity, attributed to surface physical modifications and the introduction of additional oxygen-containing functional groups. Among the polymers tested, PE and PP showed the most significant increases in both adsorption capacity and adsorption rate, with PE showing the strongest response. Among the aging treatments, persulfate/heat produced the greatest improvement in adsorption performance. Thus, the findings indicate that the sorption process is inherently complex and strongly dependent on both the polymer type and the applied aging treatment. Oxidative aging can alter the physical and chemical characteristics of MPs, thereby modifying their sorption pathways and capacities. These results underscore the importance of further research to understand these mechanisms and optimize polymer properties to enhance their performance in environmental remediation applications.

Pharmaceuticals are commonly used in human healthcare and veterinary practice; however, their proper disposal remains a problem. Recent studies (Tumrani et al. 2025; Cheng et al. 2024; Li et al. 2025) have shown that this class of compounds can be effectively adsorbed by MPs (Ge et al. 2025). An investigation of the adsorption behavior of pristine and UV-aged PVC MPs with ofloxacin (OFX) was conducted by Tumrani et al. (2025). The results demonstrated significant differences between pristine and UV-PVC, with the aged MPs reaching high absorption rates. Cheng et al. (2024) explored the aging processes of PLA and PS using the K_2_S_2_O_8_/Heat system to investigate the adsorption characteristics of tetracycline (TCs). Aging increased surface porosity and created additional active sites, which slowed the adsorption rate. When moderate oxidizers (H_2_O_2_) were applied during the aging of PET, PS, and PVC, the polymers did not lose their hydrophobicity, thereby enabling increased adsorption of levofloxacin hydrochloride (Li et al. 2025). That said, the results show that the oxidative aging of MPs profoundly alters their adsorption mechanisms and capacities for different classes of drugs, with the type of polymer and the intensity of the oxidation process being determining factors in the environmental behavior of these interactions. Table 5 presents the references found on the application of AOPs for assessing the adsorption of contaminants onto MPs.

**Table 5:** References for the application of AOPs in adsorption, desorption and sorption studies**. ICP-MS,** inductively coupled plasma mass spectrometry; **HPLC–UV-Vis**, high-performance liquid chromatography coupled to ultraviolet–visible detection; **PHA**, polycyclic aromatic hydrocarbons; **PHE**, phenanthrene; **NPAH**, nitrated polycyclic aromatic hydrocarbons

Microplastics	AOPs	Characterizations	Adsorption/Desorption	References
PE and PLA	O_3_/UV	SEM, ATR-FTIR, XPS and ICP-MS	Pb^2+^ Adsorption	(Liu et al. 2022b)
PS, PE and PP	O_3_, O_3_/H_2_O_2_	XDR, SEM, ATR-FTIR, HPLC–UV-Vis	Pesticides Adsorption	(Belé et al. 2021)
PT, PU and UF	UV and/or H_2_O_2_	XDR and FTIR	PAHs and PHE Adsorption	(Zhang et al. 2020)
PS	Fenton e H_2_O_2_	SEM and ICP-MS	Cd^2+^ Adsorption	(Lang et al. 2020)
MPs	Na_2_S_8_O_8_/PGMB	SEM, ATR-FTIR, EDX, XPS e ICP-MS	Pb^+2^ Desorption	(Ye et al. 2020)
PP, PET and PLA	K_2_S_2_O_8_	SEM, ATR-FTIR, BET, UV–VIS	Pharmaceuticals Adsorption	(Zhang et al. 2023a)
PS and PLA	K_2_S_2_O_8_/Heat	SEM, ATR-FTIR, XPS and XDR	Pharmaceuticals Adsorption	(Cheng et al. 2024)
PA	K_2_S_2_O_8_/Heat	Stereo microscopy, SEM, FTIR and BET	pharmaceuticals adsorption	(Chokejaroenrat et al. 2024)
PE, PP and PS	K_2_S_2_O_8_/Heat, UV/H_2_O_2_ and UV-PAA	BET, FTIR, SEM, XPS, XDR and DSA	NPAH adsorption and desorption	(Kong et al. 2024)
PLA	UV	SEM, FTIR, XPS and XDR	Cd adsorption	(Sun et al. 2025)
PLA	UV	SEM–EDS, BET, XDR, FTIR and XPS	Cd and Cu adsorption	(Feng et al. 2025a)
PS, PP, PET and PLA	Fe^2+^/PMS	SEM, CA, FTIR, XPS, XDR and BET	Pharmaceuticals adsorption	(Feng et al. 2025b)
PHA	K_2_S_2_O_8_/Heat	SEM, FTIR, XDR, XPS and CHN	Pharmaceuticals adsorption	(Ge et al. 2025)
PE and PLA	UV, Cl_2_ and UV/Cl_2_	FTIR, TOC, EEMs, and FESEM	Plastic additives desorption	(Lee et al. 2025b)
PVC, PS and PET	H_2_O_2_	SEM, FTIR and XPS	Pharmaceuticals adsorption	(Li et al. 2025)
PVC	UV	SEM, FTIR, XPS, DSA, 3D-EEM and XDR	Pharmaceuticals adsorption	(Tumrani et al. 2025)

## Advances and contributions of this review to microplastic pollution research

During the literature review process, 9 review articles addressing the relationship between MP pollution and the application of AOPs were identified. The previous reviews have primarily focused on the degradation and removal of microplastics, with particular emphasis on aquatic remediation technologies and oxidation kinetics (Gayathri et al. 2023; Rizwan and Bilal 2022; Shen et al. 2022; Ma et al. 2025). More recent reviews have also explored specific topics, including microplastic and antibiotic interactions, analytical protocols, and polymer degradation pathways (Li and Sui 2025; Zhang et al. 2024; Wang et al. 2023a, b, c, d). However, none of these reviews has provided a comprehensive and quantitative assessment of the multiple roles of assessed the multiple roles of AOPs across the various research domains of microplastic studies beyond degradation processes in aquatic systems. This review addresses this gap by systematically integrating the knowledge about artificial aging, degradation, organic matter digestion, MP recovery, and contaminant adsorption/desorption processes.

In addition to discussing the role of AOPs in sample preparation for organic matter digestion, a topic previously examined mainly from the perspective of microplastic recovery efficiency by Zhang et al. (2024), this review provides a comparative assessment of different oxidizing agents, considering their performance, the extent of damage induced in MPs, and the relationship between digestion efficiency and oxidation-induced surface modifications. Furthermore, it directly links the physicochemical changes occurring during artificial aging to the capacity of MPs to adsorb contaminants such as pesticides, pharmaceuticals, and metals, thereby contributing to a better understanding of their environmental behavior and fate. By synthesizing the literature published up to November 2025 and quantitatively comparing oxidant performance in terms of degradation efficiency, recovery rates, and physicochemical transformations, this review offers a comprehensive perspective on the current state and future directions of AOPs applications in microplastic research and environmental management.

The analysis of the available literature reveals distinct roles of AOPs across different stages of microplastic research. Persulfate-based systems emerge as highly effective approaches for accelerated aging, offering an alternative to conventional UV-based aging methods, whereas hydrothermal and Fenton-based treatments show considerable potential for degradation and remediation of MPs. However, the major challenge associated with these methods is their high energy demand, which limits their large-scale implementation and remains a critical issue to be addressed in future research. In sample preparation, H₂O₂ and Fenton reagents remain the most widely used oxidants, whereas NaClO has also demonstrated promising performance. Each oxidant presents specific advantages and limitations regarding organic matter removal efficiency, processing time, matrix characteristics, microplastic recovery, and preservation of particle integrity. The latter has become an increasingly important consideration in methodological studies and is expected to receive greater attention as analytical protocols continue to evolve. The evidence from aging studies further demonstrates that oxidation-induced surface modifications can substantially alter the environmental behavior of microplastics by influencing their adsorption capacity and interactions with contaminants, including pesticides, pharmaceuticals, and metals. Despite these advances, important knowledge gaps remain regarding the standardization of experimental protocols, the transformations induced by oxidation in aged PMs to understand their long-term environmental consequences, as well as the scalability of AOP-based technologies for the elimination of these particles. These challenges highlight the priorities for future research to optimization of AOP applications in MPs research and environmental management.

## Conclusions

Advanced oxidative methods, including Fenton and H_2_O_2_ variants, have been the primary methods in MPs research. Investigations into aging, degradation, and digestion have emerged as key applications of AOPs. The findings from the photodegradation tests indicated that time and the concentrations of H_2_O_2_ and DOM were essential for expediting the aging process. Among the AOPs used in aging investigations, persulfate emerged as the oxidizing agent that induced the most significant morphological and chemical changes, thereby aging these plastic particles at the fastest rate, achieving results equivalent to several years of environmental exposure. Analyses of MPs degradation in effluents reveal notable resilience against these emerging pollutants, highlighting the need for the development of more effective, robust technologies to facilitate their mineralization. Increasing the temperature via a hydrothermal method combined with the Fenton and electron-fenton reactions has proven to be a viable option. However, further development is necessary for large-scale implementation. In terms of organic matter digestion, the oxidants H_2_O_2_ and Fenton showed encouraging outcomes.

Nevertheless, NaClO proved the most efficient oxidant, causing minimal impact on MPs. The recovery tests of MPs post-digestion using Fenton and H_2_O_2_ indicated good recovery efficiency for most MPs, however, the recovery of PVC and PET particles posed difficulties due to the breakdown of PVC particles, which created new MPs, and the matrix effect that hindered the recovery of PET particles, leading to a low recovery rate. Regarding adsorption capacity, it was noted that increases in specific surface area and the number of functional groups on the MPs surface contribute to its adsorption capacity. The type of polymer, the intensity of the oxidation process, and environmental factors such as pH, ionic strength, and DOM, also affects adsorption behavior in the environment.

## Data Availability

This study is a systematic review based on previously published literature. No new datasets were generated duringthis study. The search strategy, eligibility criteria, and study selection process are fully described in the manuscript,enabling the review to be reproduced.This study is a systematic review based on previously published literature. No new datasets were generated duringthis study. The search strategy, eligibility criteria, and study selection process are fully described in the manuscript,enabling the review to be reproduced.

## Declarations

### Use of artificial intelligence

Artificial intelligence tools were used solely to improve the clarity and language of the manuscript. The authors take full responsibility for the content of the manuscript.
